# Effectiveness of road safety interventions: An evidence and gap map

**DOI:** 10.1002/cl2.1367

**Published:** 2024-01-03

**Authors:** Rahul Goel, Geetam Tiwari, Mathew Varghese, Kavi Bhalla, Girish Agrawal, Guneet Saini, Abhaya Jha, Denny John, Ashrita Saran, Howard White, Dinesh Mohan

**Affiliations:** ^1^ Transportation Research and Injury Prevention Centre Indian Institute of Technology Delhi New Delhi India; ^2^ St. Stephen's Hospital Orthopaedic Delhi India; ^3^ Department of Public Health Sciences University of Chicago Chicago Illinois USA; ^4^ Texas A & M University College Station Texas USA; ^5^ Faculty of Life and Allied Health Sciences M S Ramaiah University of Applied Sciences, Bangalore Karnataka India; ^6^ Campbell Collaboration New Delhi India

## Abstract

**Background:**

Road Traffic injuries (RTI) are among the top ten leading causes of death in the world resulting in 1.35 million deaths every year, about 93% of which occur in low‐ and middle‐income countries (LMICs). Despite several global resolutions to reduce traffic injuries, they have continued to grow in many countries. Many high‐income countries have successfully reduced RTI by using a public health approach and implementing evidence‐based interventions. As many LMICs develop their highway infrastructure, adopting a similar scientific approach towards road safety is crucial. The evidence also needs to be evaluated to assess external validity because measures that have worked in high‐income countries may not translate equally well to other contexts. An evidence gap map for RTI is the first step towards understanding what evidence is available, from where, and the key gaps in knowledge.

**Objectives:**

The objective of this evidence gap map (EGM) is to identify existing evidence from all effectiveness studies and systematic reviews related to road safety interventions. In addition, the EGM identifies gaps in evidence where new primary studies and systematic reviews could add value. This will help direct future research and discussions based on systematic evidence towards the approaches and interventions which are most effective in the road safety sector. This could enable the generation of evidence for informing policy at global, regional or national levels.

**Search Methods:**

The EGM includes systematic reviews and impact evaluations assessing the effect of interventions for RTI reported in academic databases, organization websites, and grey literature sources. The studies were searched up to December 2019.

**Selection Criteria:**

The interventions were divided into five broad categories: (a) human factors (e.g., enforcement or road user education), (b) road design, infrastructure and traffic control, (c) legal and institutional framework, (d) post‐crash pre‐hospital care, and (e) vehicle factors (except car design for occupant protection) and protective devices. Included studies reported two primary outcomes: fatal crashes and non‐fatal injury crashes; and four intermediate outcomes: change in use of seat belts, change in use of helmets, change in speed, and change in alcohol/drug use. Studies were excluded if they did not report injury or fatality as one of the outcomes.

**Data Collection and Analysis:**

The EGM is presented in the form of a matrix with two primary dimensions: interventions (rows) and outcomes (columns). Additional dimensions are country income groups, region, quality level for systematic reviews, type of study design used (e.g., case‐control), type of road user studied (e.g., pedestrian, cyclists), age groups, and road type. The EGM is available online where the matrix of interventions and outcomes can be filtered by one or more dimensions. The webpage includes a bibliography of the selected studies and titles and abstracts available for preview. Quality appraisal for systematic reviews was conducted using a critical appraisal tool for systematic reviews, AMSTAR 2.

**Main Results:**

The EGM identified 1859 studies of which 322 were systematic reviews, 7 were protocol studies and 1530 were impact evaluations. Some studies included more than one intervention, outcome, study method, or study region. The studies were distributed among intervention categories as: human factors (*n* = 771), road design, infrastructure and traffic control (*n* = 661), legal and institutional framework (*n* = 424), post‐crash pre‐hospital care (*n* = 118) and vehicle factors and protective devices (*n* = 111). Fatal crashes as outcomes were reported in 1414 records and non‐fatal injury crashes in 1252 records. Among the four intermediate outcomes, speed was most commonly reported (*n* = 298) followed by alcohol (*n* = 206), use of seatbelts (*n* = 167), and use of helmets (*n* = 66). Ninety‐six percent of the studies were reported from high‐income countries (HIC), 4.5% from upper‐middle‐income countries, and only 1.4% from lower‐middle and low‐income countries. There were 25 systematic reviews of high quality, 4 of moderate quality, and 293 of low quality.

**Authors' Conclusions:**

The EGM shows that the distribution of available road safety evidence is skewed across the world. A vast majority of the literature is from HICs. In contrast, only a small fraction of the literature reports on the many LMICs that are fast expanding their road infrastructure, experiencing rapid changes in traffic patterns, and witnessing growth in road injuries. This bias in literature explains why many interventions that are of high importance in the context of LMICs remain poorly studied. Besides, many interventions that have been tested only in HICs may not work equally effectively in LMICs. Another important finding was that a large majority of systematic reviews are of low quality. The scarcity of evidence on many important interventions and lack of good quality evidence‐synthesis have significant implications for future road safety research and practice in LMICs. The EGM presented here will help identify priority areas for researchers, while directing practitioners and policy makers towards proven interventions.

## 1 PLAIN LANGUAGE SUMMARY

### 1.1 Mapping the gaps in research evidence on the effectiveness of road safety interventions

Reducing road traffic injuries (RTI) is an important goal for all countries. Progress towards this goal requires evidence‐based evaluation of the effectiveness of various road safety measures – interventions – in improving road safety. Mapping the availability of evidence on the effectiveness of road safety measures is the first step in evaluating the ‘shape and size’ of the evidence, including its gaps.

### 1.2 What is this evidence and gap map (EGM) about?

A large majority of RTI happen in low‐ and middle‐income countries (LMICs). As many LMICs continue their development of highway infrastructure, it is important for them to be able to evaluate the effectiveness of various interventions in enhancing road safety. This can help with efficient resource allocation.

Such an evaluation requires knowing what evidence is available and where there are gaps in evidence. This EGM is the first step towards understanding what evidence is available and from where.

### 1.3 What is the aim of this EGM?

This EGM is a visual resource presenting a comprehensive overview of existing knowledge about road safety and helps identify gaps in research evidence.

### 1.4 What studies are included?

The EGM includes systematic reviews and other literature that reports impact evaluations assessing the effectiveness of road safety interventions. The studies were classified based on the target of the road safety interventions: human behaviour, road infrastructure, vehicle design, post‐crash care and institutional frameworks.

### 1.5 What is the distribution of evidence?

Human factors such as enforcement and road user education (*n* = 771), and road design, infrastructure and traffic control (*n* = 661), were the two most evaluated categories of interventions. These were followed closely by the intervention category of legal and institutional framework (*n* = 424).

The least reported interventions were in the categories of post‐crash pre‐hospital care (*n* = 118) and vehicle factors and protective devices (*n* = 111).

Among the primary outcomes, fatal crashes were reported in 1414 records and non‐fatal injury crashes in 1252 records.

Among the four behavioural or intermediate outcomes, speed (*n* = 298) and alcohol use (*n* = 206) were most frequently reported followed by the use of seatbelts (*n* = 167) and use of helmets (*n* = 66).

In terms of income groups, 96% of the studies were from high‐income countries, 4.5% from upper‐middle‐income countries, and only 1.4% from lower‐middle and low‐income countries.

There were 25 systematic reviews of high quality, four of moderate quality and 293 of low quality.

### 1.6 What do the findings of the EGM mean?

The evidence map suggests that LMICs are highly underrepresented across the studies. This has two main implications. First, the effectiveness of a safety measure in a setting dominated by cars may not translate to a setting with a mix of traffic that includes a large proportion of pedestrians and motorised two‐wheelers.

Secondly, due to this bias in regional representation, the interventions that are of greater importance in LMICs – such as those aimed at increasing helmet use and designing safer buses and cars for pedestrian safety – also remain poorly studied.

### 1.7 How up‐to‐date is this map?

The authors searched for studies up to December 2019.

## 2 BACKGROUND

### 2.1 Introduction

The World Health Organization (WHO) released its World Report on Road Traffic Injury Prevention in 2004 (Peden et al., 2004). This report focused on RTI and fatalities as a worldwide health problem and included a summary of the known risk factors associated with road traffic crashes and possible countermeasures that should be put in place to control the problem. It also pointed out that without new or improved interventions, RTI will be the third leading cause of death by the year 2020. The publication of this report spurred some national and international agencies and civil society groups to give more attention to the problem of road safety and a number of resolutions were passed by the United Nations General Assembly, World Health Assembly and the Executive Board of the WHO (WHO, 2004, 2009, 2011, 2016).

As a follow‐up, three Global Ministerial Conferences on Road Safety have been held in Moscow, Russia (19‐20 November 2009), Brasilia, Brazil (18‐19 November 2015), and in Stockholm, Sweden (19‐20 February 2020). At the close of the conference in Brasilia, the 2200 delegates adopted the Brasilia Declaration on Road Safety through which they agreed on ways to halve road traffic deaths by the end of this decade – a key milestone within the new Sustainable Development Goal (SDG) target 3.6 (Brasilia Declaration 2015). The SDGtarget for 2020 was not achieved and the total number of RTI and fatalities are still increasing in most countries of the world. The ministers and heads of delegations at the third Global Ministerial Conference in Stockholm in 2020 called for a first High‐Level Meeting of the United Nations General Assembly on Road Safety at the level of Heads of State and government to mobilize adequate national leadership and advance international and multi‐sectoral collaboration in all the areas covered by the Stockholm Declaration to deliver a 50% reduction in deaths and injuries over the next decade (i.e., by 2030) and Vision Zero by 2050. The Declaration also calls upon the WHO to prepare an inventory of proven strategies and initiatives from a wide variety of member countries that have successfully reduced fatalities in member countries and have a report readied for publication in 2024.

The WHO has also released four Global Status Reports on Road Safety in 2009, 2013, 2015 and 2018. The final one being the Global Status Report on Road Safety 2018 (GSRR18; WHO, 2018). These reports give a broad assessment of the status of road safety in ~180 countries. The data were obtained from national governments using a standardized survey form. The GSRRS18 shows that the overall global road traffic fatality rate is 17.4 per 100,000, but there is a great disparity by income and regions. Low‐ and middle‐income countries (LMICs) are reported to have the highest annual road traffic fatality rates, at 24.1 per 100 000, while the rate in high‐income countries (HICs) is lowest at 9.2 per 100,000. Over half of those who die in road traffic crashes are pedestrians, bicyclists and users of motorized two‐wheelers (MTW).

Traffic injuries have been steadily rising in LMICs, and now rank in the top 10 causes of death (WHO, 2018). RTIs have become the leading cause of death for young adults in most of these countries. While most HIC have well‐established road safety policies, many LMICs are in the process of establishing national regulatory agencies and sustainable funding streams to support large‐scale interventions that systematically address risky behaviours, trauma care, and the safety characteristics of vehicle and road infrastructure.

LMIC are also growing their rural road and highway infrastructure. National governments and international development agencies consider the expansion of road infrastructure a key strategy for economic and social development. In the last two decades, China has built a highway system that rivals that of the United States, with plans for substantial expansion (Xu & Nakajima, 2015; Yan, 2011). In India, the rapid growth of the highway infrastructure is currently underway because insufficient road transport is viewed as a key impediment to industrial growth (Ghani & Goswami, 2013). Africa, where most people do not have access to all‐weather roads, plans to expand its road network by 6–10 times by 2040 (Programme for Infrastructure Development in Africa, 2013). This increase in road infrastructure and the number of vehicles is likely to result in an increase in RTIs unless accompanied by appropriate evidence‐based road safety interventions.

The historical experience of many HICs shows that traffic injuries can be reduced despite an increase in exposure (vehicle kilometres travelled or greater network of highways). Until the 1960s, most high‐income countries witnessed an increase in their traffic injury burden. However, many of these countries successfully reduced their road death toll through road safety legislation, setting up regulatory authorities, and implementing evidence‐based interventions related to road design, vehicle safety, trauma care, and traffic enforcement (Bhalla et al., 2020). Consequently, the Decade of Action for Road Safety 2011–2020, officially proclaimed by the UN General Assembly in March 2010, and SDG 3.6, sought to save millions of lives by building road safety management capacity; improving the safety of road infrastructure; further developing the safety of vehicles; enhancing the behaviour of road users; and improving post‐crash response. Several national and international initiatives have been taken over the past decade to promote and fund road safety initiatives around the world. While there is reasonable agreement internationally on safer designs of cars for occupant safety (except locally produced vehicles like three‐wheeled scooter taxis, tuk‐tuks, jeepneys, etc.), there is a lack of evidence‐based interventions in road and infrastructure design, police enforcement and post‐crash care (Davey & Freeman, 2011; Hauer, 2019; Wilson & Gangathimmaiah, 2017).

Since the proclaimed targets of 2011–2020 Decade of Action remain far from being achieved, in October 2021, WHO kicked off the Second Decade of Action for Road Safety 2021–2030, which targets to achieve at least 50% reduction in road traffic deaths and injuries by 2030. This was declared unanimously by governments around the world through a UN General Assembly resolution. WHO and the UN regional commissions, in cooperation with other partners in the UN Road Safety Collaboration, have developed a Global Plan for the Decade of Action that aligns with Stockholm Declaration. Besides recommending actions that draw from proven and effective interventions, this plan also highlights the importance of generating evidence when it's lacking:

‘Academic and research institutions play an important role in generating evidence to help government and other actors understand (through epidemiological and risk analyses) the nature of the problem as well as to identify effective solutions and strategies (through intervention trials and implementation studies)’ (WHO, 2021).

### 2.2 Impact of RTI on society

Several high‐income countries have estimated the costs of road traffic crashes over the past three decades. The methods used and costs allocated have generated a great deal of discussion and debate, in particular, because of the difficulty of putting monetary values on pain and suffering. A study undertaken by the European Federation of Road Traffic Victims, in collaboration with the Commission for European Union, on the impact of road death and injury gives the following qualitative conclusions regarding the effect of road traffic crashes on victims (European Federation of Road Traffic Victims, 1997).

- Physical and mental impairment through road traffic injury can have long‐term effects which deny victims the ability to maintain their standard of living.
- A large proportion of the relatives of dead and disabled victims, as well as the disabled themselves, suffer psychological disorders. The worst situation is that of the relatives of the dead.
- The bereaved are the worst affected – 70% – by relationship problems, communication difficulties and sexual problems. The figure for relatives of disabled victims is 40%, and for the disabled themselves 50%. After 3 years these problems do not decrease as one would expect but worsen for each category by about 5 points.
- About 50% of the relatives of victims, and the victims themselves, state that for extended periods they consume more psychotropic products like tranquilizers, sleeping tablets, tobacco, alcohol and drugs than before the incident.
- It is sometimes reported that due to the tragedy, the relationship of the respondents with their normal social partners deteriorates.
- The capacity to enjoy life as before the crash tragically disappears for 91% of the relatives of dead victims for the first 3 years. After this period, the loss persists for long periods for 84% of them. For many, this loss will be permanent.

We have quoted from this report extensively because it is important to note that the economic costing of human tragedies can only be used as an inefficient tool to understand the overall costs of the problem.

### 2.3 Why it is important to develop the EGM

In a recent paper Hauer (2019) states:

‘Over the past two decades or so progress has been made towards evidence‐based practice. Research and researchers provided valuable tools for practitioners to use. But much of practice is still opinion‐based and the role of research remains ambiguous. The first step towards reform is to rethink and then to revamp the research‐practice relationship. The reformed relationship should be endowed with a purposeful structure, one that cures what dysfunction there is and promotes the generation of trustworthy evidence’.

Evidence‐based policy requires: (1) knowing what works, and (2) implementing what has been established to work. This EGM focuses attention on the former. Unfortunately, in the absence of local research on road‐related safety interventions, roads and highways in LMIC are being designed to safety standards of HIC without an adequate understanding of the evidence base of existing standards. For example, it is generally accepted that traffic calming measures like chicanes, road narrowing, and roundabouts are effective in reducing RTI (Bunn et al., 2003, Ewing & Dumbaugh, 2009; Rothman et al., 2014). However, the effectiveness of some of these measures in LMICs is not known. Vehicles like MTWs, which are common in LMICs, wll likely not be affected by traffic calming measures in the same way as cars. Many systematic reviews also point out the fact that evidence for road safety interventions may be available for HICs, but the same is lacking from LMICs (Mulvaney et al., 2015; Roberts & Kwan, 2003). Here, it should be emphasized that just knowing what works is not sufficient. There is an implementation gap, such that even in HICs, many standards for vehicles, roads, and policing activities are being promoted without the availability of adequate scientific evidence regarding their effectiveness (Elvik, 2017; Hauer, 2019).

SDG Target 3.6 for road safety aimed to halve the number of global deaths and injuries from road traffic accidents by 2020 (United Nations, 2017). However, considering the example of the WHO European region, despite a decrease of 8.1% in road traffic deaths between 2010 and 2013, the region may not be able to fulfil this SDG 2020 target to halve the road traffic deaths and injuries (Jackisch et al., 2015; WHO, 2018). The SDG target 11.2 seeks to provide access to safe, affordable, accessible and sustainable transport systems for all, improving road safety, notably by expanding public transport, with special attention to the needs of those in vulnerable situations, women, children, people with disabilities and older people. In order to come closer to accomplishing these targets, it is important to allocate resources to promote interventions that are effective in achieving outcomes in the context of road safety. A mapping such as that provided in the present report will provide a comprehensive overview of existing knowledge in the area of road safety and its effectiveness across the world. The map will guide programme managers to high‐quality evidence and inform targeted commissioning of future research.

### 2.4 Existing EGMs and/or relevant systematic reviews

A map of evidence maps conducted in LMICs identified no EGM conducted around transportation or related adaptive measures (Phillips et al., 2017). This EGM keeps a global focus, and includes studies on the effectiveness of road safety interventions from countries on all continents. The EGM strives to capture relevant studies conducted, and broaden the included interventions and outcomes to better reflect the state of evidence in road safety in 2019. The following systematic reviews and synthesis studies will be incorporated in the EGM after being subjected to inclusion/exclusion criteria.

### 2.5 Cochrane reviews

The Cochrane Injuries Group (https://injuries.cochrane.org/) is one of the 53 Cochrane Review Groups which are part of Cochrane, an international not‐for‐profit and independent organisation, dedicated to making up‐to‐date, accurate information about the effects of healthcare readily available worldwide. The Cochrane Injuries editorial base is located at the London School of Hygiene & Tropical Medicine and its international members include researchers, health care professionals and people using health services. The group has conducted systematic reviews on the following subtopics (numbers in parentheses represent the number of reviews):

- Injury prevention (37)
- Prevention of RTI (28)
- Pre‐hospital care of trauma (5)
- Emergency medicine (32)
- Head injury (53)
- Spinal cord injury (15)
- Chest trauma (4)
- Abdominal trauma (12)

All reviews satisfying the inclusion/exclusion criteria of this EGM have been included in this study.

### 2.6 Non‐Cochrane reviews

The following sources of systematic reviews have been reviewed and those satisfying the inclusion/exclusion criteria of the present EGM included.

a.
*Articles published in journals and grey literature* . Some examples are given below:
  1. Brieger, F., Hagen, R., Vetter, D., Dormann, C.F., & Storch, I. (2016). Effectiveness of light‐reflecting devices: A systematic reanalysis of animal‐vehicle collision data. Accident & Prevention, 97, 242–260.
  2. Decker, J. S., Stannard, S. J., McManus, B., Wittig, S. M. O., Sisiopiku, V. P., & Stavrinos, D. (2015). The impact of billboards on driver visual behaviour: A systematic literature review. Traffic Injury Prevention, 16 (3).
  3. Desapriya, E. Subzwari, S., Sasges, D., Basic, A., Alidina, A., Turcotte, K., & Pike, I. (2010). Do light truck vehicles (LTV) impose greater risk of pedestrian injury than passenger cars? A meta‐analysis and systematic review. Traffic Injury Prevention, 11 (1).
  4. Elvik, R., & Greibe, P. (2005). Road safety effects of porous asphalt: A systematic review of evaluation studies. Accident Analysis & Prevention, 37, 515–522.
  5. Fu, C., Zhang, Y., Qi, W., & Cheng, S. (2016). Effects of digital countdown timer on intersection safety & efficiency: A systematic review. Traffic Injury Prevention, 17 (1).
  6. Kim, C.‐Y., Wiznia, D. H., Averbukh, L., Dai, F., & Leslie, M.P. (2015). The economic impact of helmet use on motorcycle accidents: A systematic review and meta‐analysis of the literature from the past 20 years. Traffic Injury Prevention, 16 (7).
  7. Napolitano, L. M. (2017). Prehospital tranexamic acid: What is the current evidence? Trauma Surgery & Acute Care Open, 2, e000056. https://doi.org/10.1136/tsaco-2016-000056
  8. Roshandel, S., Zheng, Z, & Washington, S. (2015). Impact of realtime traffic characteristics on freeway crash occurrence: Systematic review and meta‐analysis. Accident Analysis & Prevention, 79, 198–211.
  9. Unsworth, C.A., & Baker, A. (2014). Driver rehabilitation: A systematic review of the types and effectiveness of interventions used by occupational therapists to improve on‐road fitness‐to‐drive. Accident Analysis & Prevention, 71, 106–114.
b.
*The Handbook of Road Safety Measures* (Elvik et al., 2009). This handbook gives state of the art summaries of current knowledge regarding the effects of 128 road safety measures. It covers all areas of road safety including traffic control, driver training, publicity campaigns, police enforcement, and general policy instruments.

### 2.7 Synthesis of studies

The *SafetyCube* project (https://www.roadsafety-dss.eu/#/) synthesized existing knowledge on road safety risk factors and countermeasures in comprehensive synopses. These are listed as per risk factor/measure, colour code (assigned to reflect the strength of evidence on the effect of the risk factor or measure), and the road safety area concerned (behaviour, infrastructure, vehicle).

## 3 OBJECTIVES

The aim of the EGM is to identify, map, and describe the existing evidence on the effectiveness of interventions to improve road safety across all countries. The EGM aims to direct future research and discussions based on systematic evidence towards the approaches and interventions which are most effective in the road safety sector. This could enable the generation of evidence for informing policy at global, regional or national levels.

The objectives of this EGM are:

1. To identify existing evidence from all effectiveness studies and systematic reviews related to road safety interventions
2. To identify existing gaps in evidence where new primary studies and systematic reviews could add value.

### 3.1 Evidence and gap maps: Definition and purpose

This EGM aims to gather, present and describe the existing evidence on the effectiveness of interventions to improve road safety globally. Unlike a systematic review, an EGM only identifies what evidence exists and does not summarise or analyze the evidence itself.

## 4 EGM FRAMEWORK

### 4.1 Scope

Road safety interventions are described as the interaction of three components in light of Haddon's matrix (Haddon, 1968): human, vehicle and environment separated into pre‐crash, crash and post‐crash phases. This has been strengthened further over the past two decades by focussing on the Safe Systems approach which also includes strengthening of institutions and other road safety policy measures (Bliss & Breen, 2009). The interventions included in the EGM here focus mainly on the impact of safety interventions on road traffic‐related injuries and deaths as the primary outcome and selected safe road‐user practices as the intermediate outcome.

There are several modes of transport that are widely used in LMICs such as motorcycles and three‐wheelers, resulting in a much more heterogenous traffic mix. However, only a limited literature is available on the effectiveness of various interventions on road safety in such heterogeneous traffic. This EGM covers all aspects of road safety interventions except car‐design affecting car occupant safety. Safety standards for car design including crash worthiness standards have been evolving since 1970s. The UNECE WP.29 (United Nations Economic Commission for Europe, 2012) vehicle safety standards and New Car Assessment Programme (Global NCAP, 2017; Hobbs & McDonough, 1998) are also attempting to harmonize vehicle standards internationally. There is a general consensus on car technologies that work and don't work internationally, and car designs around the world are converging to similar international standards. Safer car designs for occupant safety are also influenced by the market because of car safety ratings announced by agencies like NCAP. However, infrastructure design, safety policies and enforcement are not subject to market mechanisms in the same way. Hence, car design interventions that only benefit car occupants have been excluded from this EGM.

The scope of this EGM is to look at studies in the road safety sector from all countries, and classify relevant studies which talk of the effectiveness of interventions primarily in terms of traffic crash injuries and deaths.

The interventions adopted in this EGM are classified into five broad categories: human factors; vehicle factors and protective devices; road design; infrastructure and traffic control; post‐crash pre‐hospital care; and legal and institutional framework. They are described as follows:

- Human factors: These cover all interventions including any human factor or road user behaviour that leads to the occurrence or consequence of RTI.
- Vehicle factors and protective devices: These are mainly focused on the design of different vehicle modes except cars (for occupant protection) and protective equipment which may lead to a reduction in injuries.
- Road design, infrastructure and traffic control: These interventions cover various types of infrastructure (geometry, traffic control, etc.) present on different categories of roads (urban and rural roads) and are critical factors affecting road traffic injury.
- Post‐crash pre‐hospital care: These pre‐hospital interventions (e.g., at the roadside, in an ambulance etc.) aims to reduce the severity of injury consequences once a road traffic crash has occurred.
- Legal and institutional framework: These mainly focus on insurance policies, vehicle taxes, fuel and road pricing, central government, research institutions and laws addressing road traffic injury.

The protocol for this EGM was preregistered with Campbell: Mohan, D, Tiwari, G, Varghese, M, et al. (2020). PROTOCOL: Effectiveness of road safety interventions: An evidence and gap map. *Campbell Systematic Reviews*, 16, e1077. https://doi.org/10.1002/cl2.1077.

### 4.2 Conceptual framework

**Figure 1:**
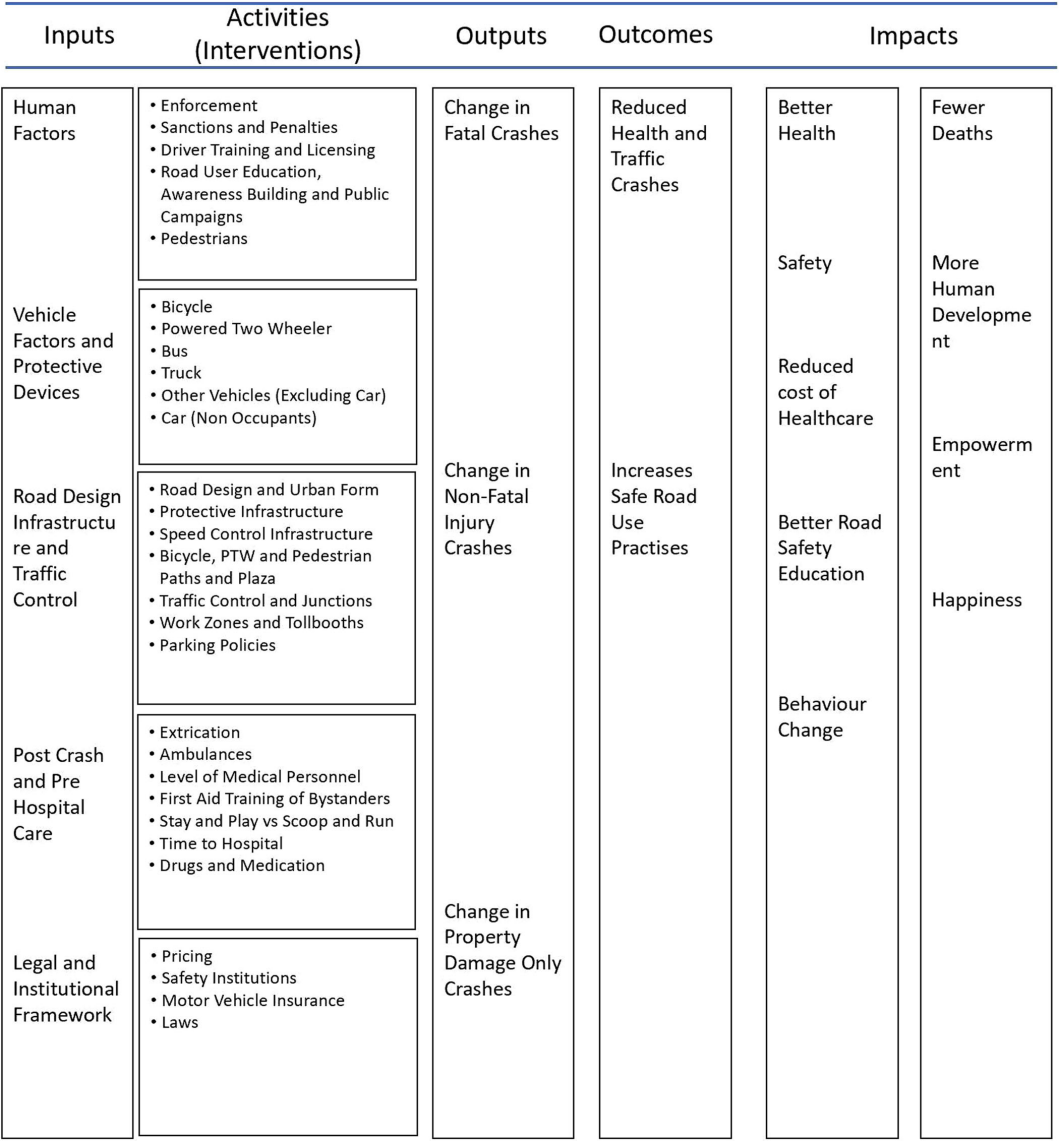
Conceptual framework for the EGM. Source: Authors based on White and Gunnarson (2008).

The conceptual framework links road safety interventions with the outcomes and impacts along the causal chain (Figure 1). The conceptual framework shows the causal chain through which the inputs are turned into final societal impacts, through activities, outputs and outcomes.

It is to be noted that the links in the causal chain may include other supporting factors. For example, a reduction in injuries and deaths due to police enforcement results from a legal and institutional framework that lays down laws and develops the capacity for enforcement and enforcement resulting in safer road user behaviour.

A scientific approach to control RTIs evolved in high‐income countries over the last 50 years or so. The main concept behind the evolving strategies rested on the understanding that we must move away from finding fault with victims and instituting retribution systems to a more reasoned approach that deals with systemic improvements and finding solutions that by and large do not put an extra burden on road users. It was understood that RTIs result from a complex interaction of sociological, psychological, physical and technological phenomena, but, since injuries result from an exchange of energy between the environment and the human body, it is possible to develop safety policies and strategies in a scientific and comprehensive manner. This will require a shift to an approach with a result focus that aims to eliminate road deaths and serious injuries by adopting interventions that are more likely to succeed in aiming for Vision Zero (Svensson, 2018).

The intervention categories in this EGM are adapted from the Haddon's Matrix which presents the typologies of road traffic crashes in time and space (Haddon, 1972). The time category is divided into three mutually exclusive categories comprising interventions that (i) prevent the occurrence of a crash, (ii) prevent or reduce the severity of injuries during a crash, and (iii) reduce the possibility of a negative outcome after the crash. The space categories include those interventions that are associated with human beings, vehicles and the rest of the environment. The last category includes road infrastructure, legislation, policing and the role of institutions. The systems approach as detailed by Bliss and Breen (2009) further elaborates the role of different institutions and inputs included in the environment category in Haddon's Matrix.

One of the reasons that road safety interventions were not particularly successful in the first 60 years of motorization (1910–1970) in any part of the world was because policy makers and researchers could not measure the effectiveness of various interventions in terms of fatalities, injuries or crashes prevented (O'Neill & Mohan, 2020). Subsequent research indicated that some interventions may have been based on theoretical beliefs of their effectiveness but were not so in real use and others may produce behaviour change but not found to be effective in the real world of traffic. For example, recent systematic reviews indicate that the following interventions do not result in fewer RTI and outcomes:

- Advanced training in trauma life support for ambulance crews (Jayaraman et al., 2014)
- Interventions in the alcohol server setting for preventing injuries (Ker & Chinnock, 2008)
- Spinal immobilization for trauma patients (Kwan et al., 2004).
- Motorcycle rider training for the prevention of road traffic crashes (Kardamanidis et al., 2010).
- Post‐licence driver education for the prevention of road traffic crashes (Ker et al., 2005).
- Safety education of pedestrians for injury prevention (Duperrex et al., 2003).
- School‐based driver education for the prevention of traffic crashes (Roberts & Kwan, 2003).

Researchers in the field of traffic safety have been aware of the existence of counterintuitive results in their area of work for more than four decades. The fact that many interventions do not result in reductions in RTIs is mainly because most studies only measure intermediate outcomes like change in behaviour or knowledge and not the actual results in the field. For example, a systematic review on safety education by Duperrex et al. (2002) concludes that ‘*Pedestrian safety education can result in improvement in children's knowledge and can change observed road crossing behaviour, but whether this reduces the risk of pedestrian motor vehicle collision and injury occurrence is unknown*’. Hauer (2019) goes further and states that ‘*Issues of concern are the lightness with which decisions affecting road‐user safety can be based on opinion that is unsupported by evidence…that questionable results can be given a ring of consensual truth, and that the questions which research asks and what findings get published are at times influenced by external interest*’.

In this EGM, importance has been given to primary outcomes as there is little agreement among researchers on the relationship between behavioural/knowledge changes and reductions in traffic crashes. Therefore, studies are included only if the outcome is measured in changes in RTI (fatal, injury only) or use of protective devices (helmets and setabelts), changes in vehicle speeds, or drinking and driving.

## 5 METHODS

### 5.1 Criteria for including and excluding in this EGM

#### 5.1.1 Search sources

To conduct a comprehensive search of impact evaluation studies and systematic reviews, we developed a search strategy to cover all relevant academic databases and grey literature from organizational websites (listed in Supporting Information: Appendix 1), in consultation with our advisory team which includes several experts from this field. The final search string created is used in searching multiple sources. The complete search strategy was validated by an information retrieval specialist at 3ie, John Eyers. After the protocol had been peer‐reviewed and approved by Campbell Collaboration, the searches were conducted and all the studies, irrespective of their country, language or status of publication, were included for screening.

We searched for impact evaluations published in SafetyLit (https://www.safetylit.org/), PubMed (https://www.ncbi.nlm.nih.gov/pubmed/), TRID (https://trid.trb.org/), Web of Science database, EMBASE, Cochrane Injuries Group's Specialised Register, and Cochrane Central Register of Controlled Trials (CENTRAL). We searched for systematic reviews in Epistemonikos (https://www.epistemonikos.org/), Cochrane Library, Campbell Library, 3IE, and EPPI centre. We searched for literature using online repositories of organizations that are known to produce or keep depositories. These included SafetyCube, IIHS, SWOV Institute For Road Safety Research, World Health Organization websites (WHO), Asian Development Bank (ADB), UK Department for International Development (DFID), Centre for Global Development (CGD), International Development Research Centre (IDRC), Australian Road Research Board (ARRB), UN Road Safety Collaboration (UNRSC), Information and Technology Centres for Transport and Infrastructure – CROW (Netherlands), Danish Council for Road Safety Research, US National Highway Traffic Safety Administration (NHTSA), Swedish National Roads Administration, Institute of Transport Economics (TOI), Transportation Research Board (TRB), Transport Research Laboratory (TRL), VTI Swedish National Road and Transport Research Institute, VTT Finland, the Organisation, OECD Joint Transport Research Centre's International Transport Research Documentation (ITRD) database), European Transport Safety Council (ETSC), World Conference on Transport Research Society (WCTR), International Research Council On Biomechanics Of Injury (IRCOBI), and East Asian Science, Technology and Society: An International Journal (EASTS). Finally, we backreferenced the relevant systematic reviews and impact evaluation studies.

We used the EPPI‐Reviewer 4 software (Thomas et al., 2010) for screening and coding, and the EPPI‐Mapper (Digital Solution Foundary and EPPI‐Centre, 2020) for generating the map. EPPI‐Reviewer and EPPI‐Mapper are developed by the EPPI‐Centre at the Social Science Research Unit of the UCL Institute of Education, University of London, UK (http://eppi.ioe.ac.uk/cms/Default.aspx?alias=eppi.ioe.ac.uk/cms/er4). The results of the searches were uploaded in EPPI Reviewer 4. The grey literature and additional references were added manually to the software and the duplicates were managed using the built‐in functionality of EPPI Reviewer 4.

### 5.2 Types of evidence

The EGM included impact evaluations and systematic reviews of the effectiveness of road traffic injury interventions. Impact evaluations were defined as evaluations of intervention or field experiments that used quantitative approaches applied to experimental or observational data to measure the effect of an intervention relative to a counterfactual representing what would have happened to the same group in the absence of that intervention. Impact evaluations may also test different intervention designs. We included both completed and on‐going impact evaluations and systematic reviews. To capture the latter, we included prospective study records in trial registries or protocols when available.

Our categorization of study design is based on Elvik (2008) who reviewed the strengths and weaknesses of the various study designs that have been used for impact evaluations of road traffic injury interventions. We used the following classification:

1. Randomised control trials
2. Before‐and‐after studies
3. Case‐control
4. Time series
5. Cross‐sectional studies
6. Systematic reviews

Within case‐control, as suggested by Elvik (2008), we also included studies that used the double‐pair comparison method developed by Evans (1986) to evaluate the effects of seat belts and helmets. In these studies, both case and control individuals are involved in the same accident. Within cross‐sectional studies, we only included comparative studies controlling for confounding by stratification and studies comparing between units with and without intervention. We exclude cross‐sectional studies using multivariate regression methods. We exclude all theoretical, modelling, simulation or laboratory studies. Additionally, we excluded all studies using self‐reported crash outcomes. We excluded qualitative studies.

### 5.3 Inclusion decisions

State how inclusion decisions will be made (i.e., from search results to included studies), clarifying how many people will be involved and whether they will work independently (ER33).

### 5.4 Types of interventions/problem

The main intervention categories are based on the 5 pillars of road safety (WHO, 2011) and these five categories are then subdivided into multiple subcategories. These different intervention categories are shown in Table 1.

**Table 1:** Intervention categories of the EGM with example.

Intervention category	Subcategory	Examples
Human factors	Enforcement	1. Speed and red‐light enforcement‐ Manual policing Speed cameras: Cameras that are positioned at a specific location to take pictures of vehicles exceeding speed limits Red light Camera: Cameras that automatically detect and record red light running vehicles at intersections 2. Drink‐driving/drug enforcement: Driving with alcohol or drugs in the blood stream 3. Seat‐belt enforcement: Enforcement of legislation requiring the fitting of seat belts to motor vehicles and the wearing of seat belts by motor vehicle occupants to be mandatory 4. Helmet enforcement: A helmet is a form of protective gear worn to protect the head during an impact. The intervention includes enforcement of laws for wearing helmets while driving 5. Cell phone use: Mobile phone use while driving 6. Other offence enforcement: Enforcement of laws preventing offensive driving, for example, reckless driving 7. Vehicle inspection: Procedure mandated by national or subnational governments, in which a vehicle is inspected to ensure that it conforms to regulations governing safety
	Sanctions and penalties	1. Fines: A fine imposed for disobedience with the driving law or with rules and regulations aimed to reduce RTI 2. Imprisonment: It is a stringent form of sanction for disobedience with the driving laws or with rules and regulations 3. License demerit points: The system in which a driver's licensing authority, police force, or other organization issues cumulative demerits, or points to drivers on conviction for road traffic offenses. Points may either be added or subtracted, depending on the particular system in use. 4. License suspension: Temporary withdrawal of the right to drive a motor vehicle 5. Re‐education/Retraining programme: The programme in which drivers found committing any driving offence are required to take a retraining programme which may also be an alternative to prosecution
	Driver training and licensing	1. Driver education and training: Training drivers for skill acquisition, decision making whiledriving or risk mitigation in context of reducing incidence of RTI. 2. Driver license age: The age at which a person may obtain a driver's license to lawfully drive a motor vehicle on public roads. 3. Driving test: The tests to evaluate person'sknowledge on driving related rules and laws and to assess the person's ability to drive (hazard perception, Vision tests, health requirements) 4. Graduated driving licenses: The systemdesigned to provide to provide novice drivers of motor vehicles with driving experience and skills gradually over time in low‐riskenvironments. 5. Screening by employer (driving record, health, star rating)
	Road user education, awareness building and public campaigns	1. General, road use (children, adults, all) 2. Alcohol use 3. Road signs 4. Helmet use, Seatbelt use
	Pedestrian	1. Reflective clothing for improved visibility of pedestrians 2. Pedestrian detection systems
Vehicle factors and protective devices *(other than car occupant protection technologies)*	Bicycle	1. ABS (Anti‐lock braking system) and combined brakes, Emergency braking, speed limiters: The systems designed to prevent the problems occurring when wheels lock 2. Daytime running lights: Automotive lighting and bicycle lighting device on the front of a motor vehicle or bicycle, automatically switched on when the vehicle is in drive. 3. Reflective material: clothing worn that has highly reflective properties or a colour that is easily discernible from any background 4. Protective clothing: Clothing designed to mitigate risk of injury when rider is exposed to injury through contact with other objects 5. Helmets: Devices that reduces injuries by providing additional impact and abrasion protection to the head of a wearer in the event of a crash 6. Under‐run guards: The Under‐run protection bar enables small and large vehicles to make bumper‐to‐bumper contact thus preventing the smaller and lower vehicles to ‘ride under’ the large vehicles especially trucks 7. Active protection: Autonomous Emergency Braking AEB, Electronic Stability Control (ESC) 8. Pedestrian protection: The use of vehicle design concepts that reduce the likelihood of injuries to pedestrians in the event of a car‐pedestrian crash. 9. Vehicle Design other than occupant protection technologies
	Motorised two‐wheeler	
	Cars (non‐occupant)	
	Bus	
	Truck	
	Other vehicles	
Road design, infrastructure and traffic control	Road design and urban form	1. Lane width, number of lanes 2. Shoulder: Designing the surface immediately beyond the carriageway edge line 3. Median: Design of the physical separation between opposing traffic streams 4. Road lighting: The application of illumination systems along roadways, primarily for the purpose of improving safety by increasing visibility of roadside hazards and by reducing the effects of glare from other light sources in the visual environment, such as vehicle headlamps. 5. Black spot treatment: A systematic and scientific identification of abnormally high accident sites or hazardous road locations and a remedial process that aims to develop appropriate and cost‐effective treatments for such sites. 6. Urban form: The physical patterns, layouts, and structures that make up an urban centre are collectively called the urban form
	Protective Infrastructure	1. Guard rails: Design of longitudinal highway barriers designed to reduce the impact of Run‐off‐road collisions 2. Crash cushions: A device intended to reduce the damage to structures, vehicles, and motorists resulting from a motor vehicle collision. These are designed to absorb the colliding vehicle's kinetic energy. 3. Roadside safety treatment: This aims to remove particularly dangerous and sight reducing obstacles from the roadside and give drivers greater opportunity to regain control in the event of run‐off the road, for example, flattening side slopes, increasing distance between edge and fixed obstacles and removal of such obstacles
	Speed control infrastructure	1. Speed limits: Road speed limits are maximum speed permitted by legislation on a given stretch of road. They are indicated on a traffic sign 2. Traffic calming: Measures taken to reduce traffic volume and/or speed (e.g., bumps, humps, other raised pavement areas, street closures, traffic diversion etc.) 3. Speed reducing devices: Physical obstructions meant to slow down vehicles
	Bicycle, MTW and pedestrian paths	1. Bicycle lanes 2. MTW lanes 3. Footpaths 4. Footbridges 5. Pedestrian tunnels
	Traffic control and junctions	1. Road markings and signs (variable signs, zebra crossings, edge line rumble strips, chevrons) 2. Signalized junctions 3. Non‐signalized junctions 4. Roundabouts
	Work zones and tollbooths	1. Work zones: A work zone is an area where roadwork takes place and may involve lane closures, detours and moving equipment. 2. Toll roads: The roads for which fee or toll is assessed for passage. The toll is collected by toll booths, plazas etc. 3. Road maintenance: The maintenance of roads to keep them in good condition by carrying out scheduled repairs and reinforcement work
Post‐Crash Pre‐Hospital Care	Extrication	1. Road and helicopter ambulances including medical equipment 2. Advance life support and advance trauma life support systems in ambulances 3. Basic life support and pre‐hospital trauma life support training given to passer byes, first responders etc. 4. Time to primary trauma care 5. Stabilization of crash victims before reaching primary trauma care
	Ambulances (Road and helicopter) (including equipment)	
	Level of medical personnel	
	First aid training of bystanders	
	Stay and play vs. Scoop and run	
	Time to hospital	
	Drugs and medications	
Legal and institutional framework	Pricing	1. Vehicle taxes: Taxes imposed on vehicles based on engine capacity, sitting capacity, cost price, etc. 2. Road pricing: The charges levied on road users to discourage use of certain vehicles, fuel sources or use of busy roads at certain times. 3. Congestion Pricing: A system of surcharging road users that are subject to congestion through excess demand during peak hours 4. Fuel pricing: Change in fuel prices
	Safety institutions	1. Central agencies 2. Research institutions
	Motor vehicle insurance	1. No fault incentive for automobile drivers 2. Financial incentives in insurance for no crash history of the driver. a. Helmet law
	Laws and policy	Regulations and policy mandates like 1. Seatbelt law 2. Vehicle protective technologies such as antilock braking systems, daylight running lights etc.

For the Post‐Crash and Pre‐Hospital care, the approach that was taken was slightly different from the rest of the studies mapped in the EGM. It should be mentioned that some of the interventions observed in these studies can in theory be performed in a pre‐hospital setting, but owing to limitations in ambulance infrastructure, cost limitations, or non‐viability of specialists to execute the interventions, these interventions to our knowledge have not been implemented anywhere in the world. A prime example of this is FAST (Focused Assessment with Sonography for Trauma), which is an important triaging tool used in trauma centers and emergency departments across the world.

### 5.5 Type of population

This EGM covers all road users, i.e., both motorised and non‐motorised (including pedestrians and cyclists), with the exception of car occupants affected by car safety technologies as this has been extensively covered in literature in the past.

### 5.6 Types of location/situation

We did not have a specific criteria related to these categories.

### 5.7 Types of outcome measures

The evidence is plotted on the map based on the following outcome categories‐

- Fatal crashes
- Non‐fatal injury crashes
- Change in the use of seat belts
- Change in the use of helmets
- Change in speed
- Change in alcohol/drug use

### 5.8 Types of settings (as applicable)

Where applicable, briefly describe the types of settings that will be included and excluded.

### 5.9 Screening and study selection

Studies were screened in two phases, first from the SafetyLit database, and then from the remaining databases listed in Supporting Information: Appendix 1. SafetyLit is the largest database for such literature and indexes around 70% of road safety journals. The screening choices of the SafetyLit database were also used to train a machine learning classifier (MLC) to speed up the screening for the other databases. This process is discussed later.

All the studies were screened in 2 stages, first by title and abstract, and then by downloading and screening the full text. In both stages of screening the studies were assigned independently to two reviewers who entered their screening decisions individually. Next the two screening decisions were reconciled by a senior researcher in the team. An exception was made for the senior members of the team wherein the studies assigned to them were not simultaneously reviewed by another member of the team, and their decision was taken as final. For the second stage, i.e., screening on full text, the PDF versions of the different studies were downloaded and uploaded to EPPI Reviewer. Full‐text screening was done in a similar manner as title and abstract screening.

For the second phase the results of the manual screening done for the SafetyLit database studies were used to train the MLC functionality in EPPI Reviewer 4.0. The MLC was used only for the title abstract level screening and the true criterion was set as exclusion at a relatively low classification threshold of 70%. Out of the studies excluded by the classifier 200 randomly selected studies manually checked for any false positive results. The studies included by the classifier were then manually screened similar to how the phase 1 studies were screened for title and abstract. A total of 54,276 studies were screened by the classifier, out of which 44,209 studies were excluded and the remaining 1067 studies were manually screened

### 5.10 Data collection and analysis

#### 5.10.1 Data collection process

The coding form (Supporting Information: Appendix 2), based on the framework and PICOS, has been used for data extraction. Data extracted from each study includes bibliographic details, intervention types and descriptions, outcome types and descriptions, study design, context/geographical information and details on the comparison group.

The coding of systematic reviews is based on the characteristics of the included studies in the review which meet the PICOS for this map.

thThis tool was piloted to ensure consistency in coding and resolve any issues or ambiguities. A single researcher has conducted the data extraction for each study; however, all coders were trained on the tool before starting and a sample was double‐coded to check for consistency.

We used EPPI Reviewer software to extract descriptive data from all studies meeting our inclusion criteria.

#### 5.10.2 Tools for assessing risk of bias/study quality of included reviews

Since systematic reviews are often used for decision making, we appraised the methodological quality of systematic reviews using the AMSTAR‐2 (Assessing the Methodological Quality of Systematic Reviews) checklist (Shea et al., 2017) in duplicate by two reviewers

The 16 items in AMSTAR2 cover:

1. PICOS in inclusion criteria,
2. Ex‐ante protocol,
3. Rationale for included study designs,
4. Comprehensive literature search,
5. Duplicate screening,
6. Duplicate data extraction,
7. List of excluded studies with justification,
8. Adequate description of included studies,
9. Adequate risk of bias assessment,
10. Report sources of funding,
11. Appropriate use of meta‐analysis,
12. Risk of bias assessment for meta‐analysis,
13. Allowance for risk of bias in discussing findings,
14. Discussion and analysis of heterogeneity,
15. Assessment of publication bias,
16. Report and potential source of conflicts of interest.

Seven domains can critically affect the validity of a review and its conclusions (critical items 2, 4, 7, 9, 11, 13, and 15). The study's overall confidence ratings of the quality are high if there is no more than one noncritical weakness, medium if there is no critical weakness but more than one noncritical weakness, and low if there are one or more critical weaknesses.

Here it should be noted that the quality appraisal tool AMSTAR 2 is an extension of AMSTAR, which only appraised RCTs. However, AMSTAR 2 includes non‐randomized studies, that are common in transportation safety research.

We did not critically appraise the quality of the included impact evaluations but extracted and documented data on study design.

#### 5.10.3 Stakeholder engagement

The EGM framework was developed through consultation with an Advisory Group nominated for this project, and discussions with the council members of the Independent Council for Road Safety International (ICoRSI). We hosted a mid‐project workshop at Karolinska Institutet (Stockholm, Sweden) as a pre‐event of the 3rd Global Ministerial Conference on Road Safety on 18 Feb 2020, where we presented emerging findings and sought feedback. This public meeting was attended by senior researchers and practitioners from across the world and a variety of international institutions. The final EGM results were presented at a public workshop held at IIT Delhi on 7 September 2022.

### 5.11 Analysis and presentation

#### 5.11.1 Report structure

Describe the outline and framework for the report and map respectively, specifying the dimensions of the map and sections, tables and figures to be included in the report.

#### 5.11.2 Presentation

The EGM has two primary dimensions: interventions (rows) and outcomes (columns). Additional dimensions are:

1. Income: Low‐income countries, lower‐middle income countries, upper‐middle income countries, and high‐income countries (according to World Bank 2020 fiscal year classification).
2. Region: South Asia, Sub‐Saharan Africa, East Asia and Pacific, Europe and Central Asia, Latin America and Caribbean, Middle East and North Africa, North America (based on the World Bank classification).
3. Systematic review quality: low, moderate, high, using the AMSTAR 2 appraisal
4. Type of primary study: Before‐After with control, Case‐Control, Cross‐Section analysis, Experiments, Time Series with control.
5. Population: Pedestrian, driver, passenger, all types of motorized and non‐motorized vehicles.
6. Age: The age group of the target population including 0–5 years; 6 years ‐Licensing Age; Licensing Age – 65 years; >65 years.
7. Road type: Urban open and restricted access roads, rural open and restricted access roads.

In the hard copy of the EGM, multiple 2 × 2 representations of the EGM are reported. In the online version, selected additional dimensions are possible to use as a filter and include references to included studies.

## 6 RESULTS

### 6.1 Search results

#### 6.1.1 Search hits

The search yielded 217,801 results from all sources from which 117,894 duplicates were eliminated by a built‐in tool in EPPI Reviewer to manage duplicates. Due to this large number of search hits obtained, text mining was used on title and abstract to narrow down the list.

#### 6.1.2 PRISMA

**Figure 2:**
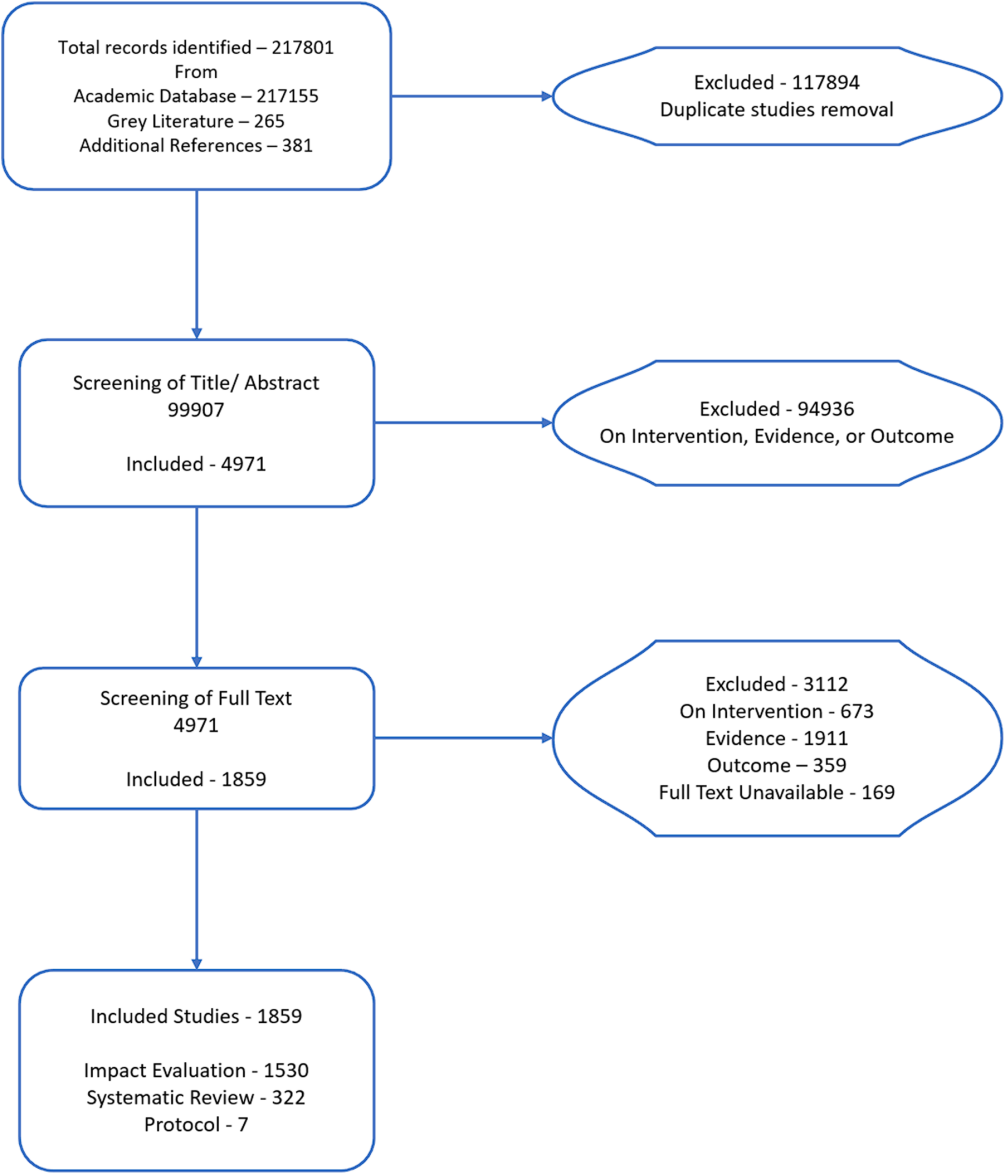
PRISMA of the road safety EGM.

The PRISMA for the EGM is shown in Figure 2 below.

### 6.2 Excluded studies

We excluded studies through full‐text review if they did not report the following as mentioned in the inclusion criteria: (a) outcome, (b) intervention, and (c) evidence. For example, based on outcome, we excluded Wahl et al. (2010) as the authors did not report outcome in terms of fatalities or injuries. Based on intervention, we excluded Ackery et al. (2012) as the authors did not report any of the intervention type shown in Figure 1. We excluded Bambach et al. (2013) as the study did not use any of the study designs listed in the inclusion criteria.

### 6.3 Synthesis of studies included

**Figure 3:**
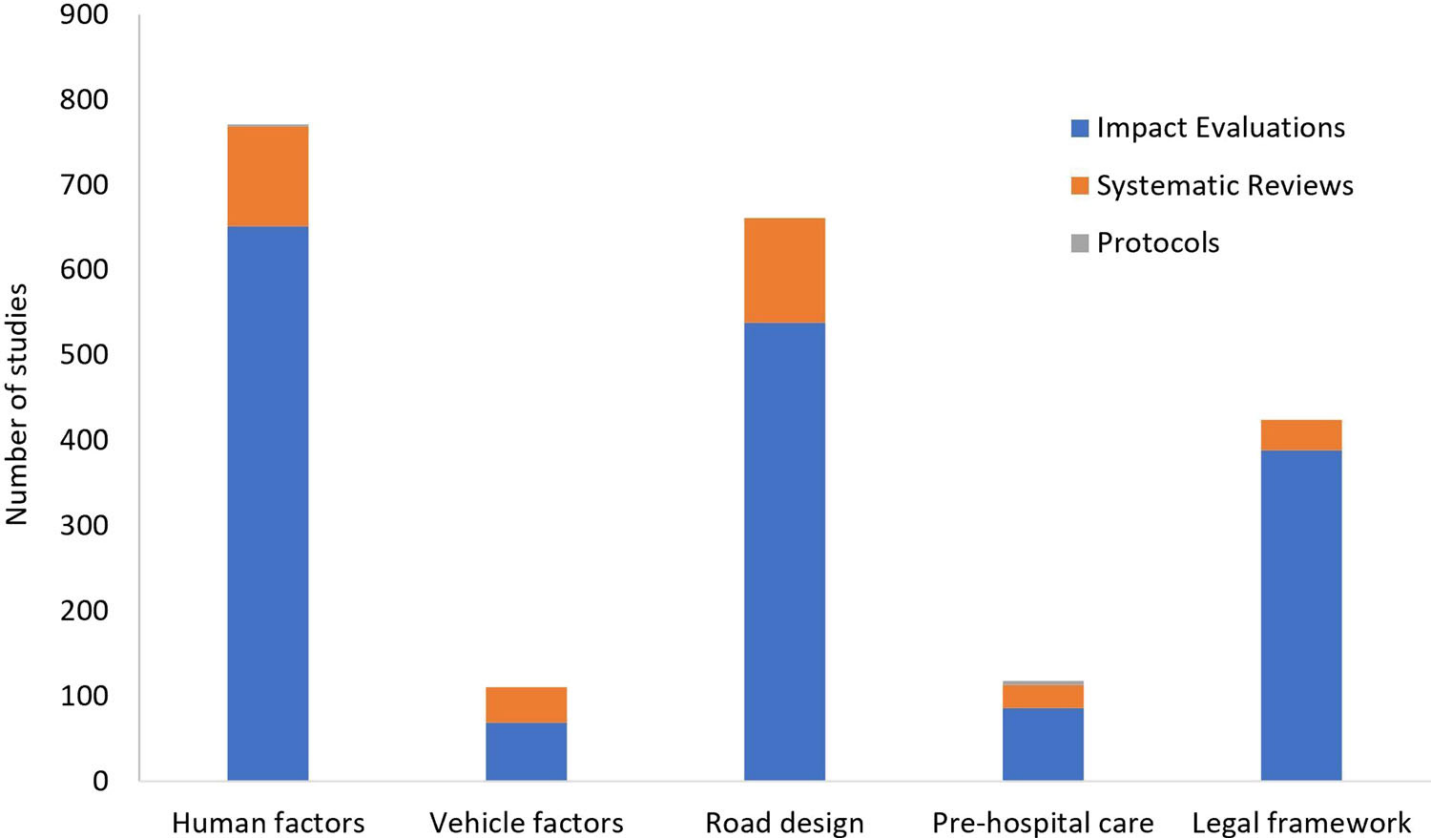
Number of studies for different types of interventions.

**Figure 4:**
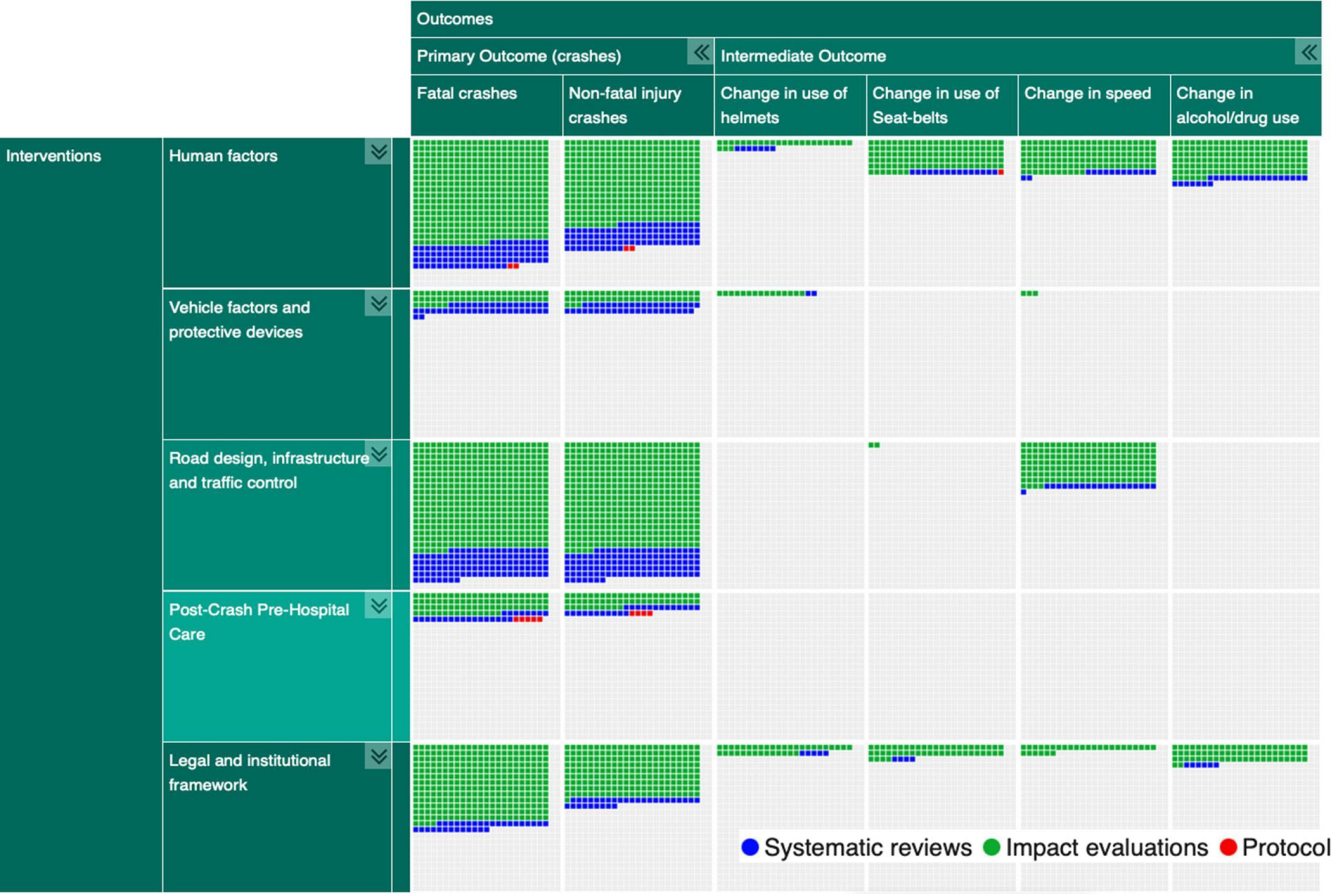
Number of impact evaluations by income category.

**Figure 5:**
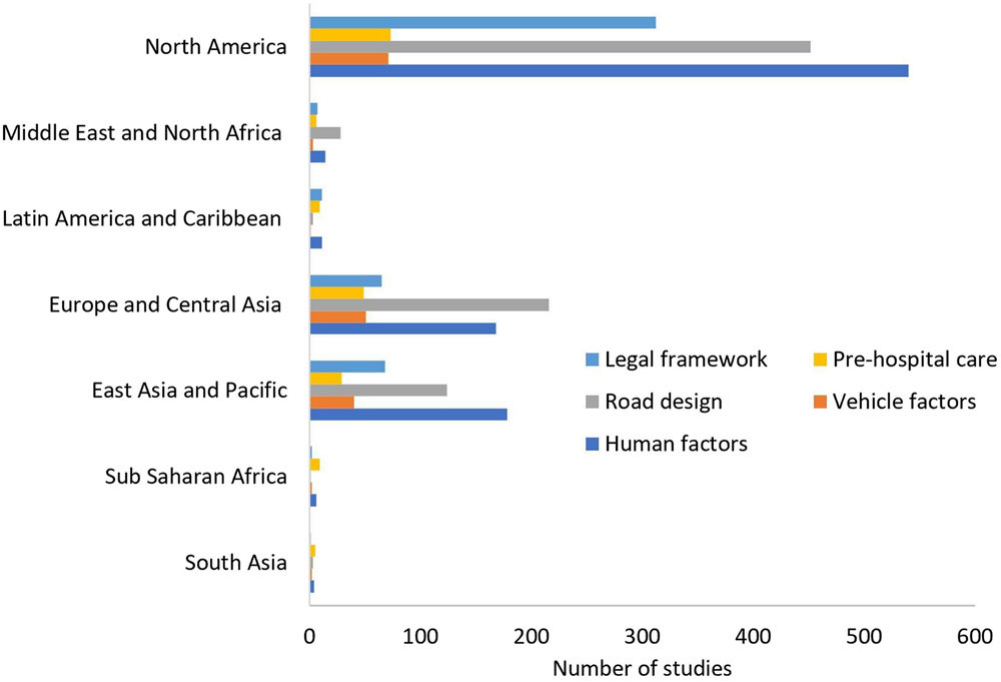
Number of studies by World Bank region.

**Figure 6:**
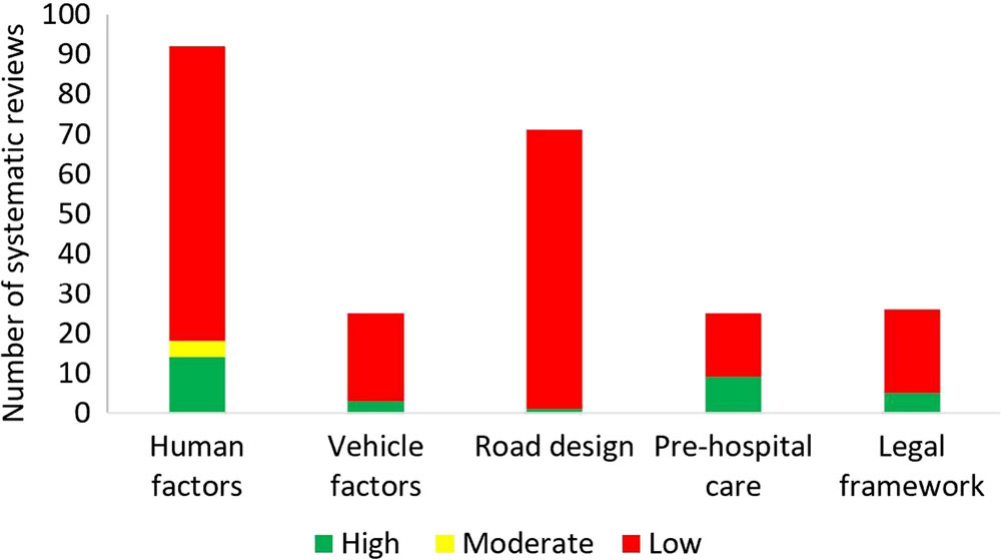
Number of systematic reviews by quality.

The EGM includes both completed and ongoing systematic reviews, completed impact evaluations and grey literature systematically searched from academic databases and organization websites. We did not limit the search by language, date and status of publication. We identified 1859 studies of which 322 are systematic reviews, 7 are protocol studies and 1530 are impact evaluations (Table 2, Figures 3 and 4). The two categories of interventions – human factors (*n* = 771) and road design, infrastructure, and traffic control (*n* = 661) were most commonly reported, followed by legal and institutional framework (*n* = 424), and the least reported were post‐crash pre‐hospital car (*n* = 118) and vehicle factors and protective devices (*n* = 111). Fatal crashes as outcomes were reported in 1414 records and non‐fatal injury crashes in 1252 records. Among the four intermediate outcomes, speed was most reported (*n* = 298) followed by alcohol (*n* = 206) and use of seatbelts (*n* = 167), and use of helmets (*n* = 66) was least reported. Ninety‐six percent of the studies were reported from high‐income countries, 4.5% from upper middle‐income countries, and only 1.4% from lower middle and low‐income countries (Figures 4 and 5). Among the geographies, North America has the maximum number of records (*n* = 1269). The next highest is Europe and Central Asia (*n* = 504) followed by East Asia and Pacific (*n* = 396). The least number of records are from the regions of South Asia, Sub‐Saharan Africa, Latin America, Caribbean, Middle East and North Africa, with a combined total of 106 records. There were 17 systematic reviews with high quality, 4 with moderate quality, and 293 with low quality (Figure 6).

**Table 2:** Number of evaluation studies by study design.

Study design	Human factors	Vehicle factors	Road design	Pre‐hospital care	Legal framework
Before and after	380	16	401	5	178
Case‐control	45	31	52	28	15
Cross‐section	11	12	45	15	20
Experiments	45	2	8	33	5
Time‐series	170	8	32	5	171
Systematic review	120	42	123	32	35

### 6.4 Human factors

**Figure 7:**
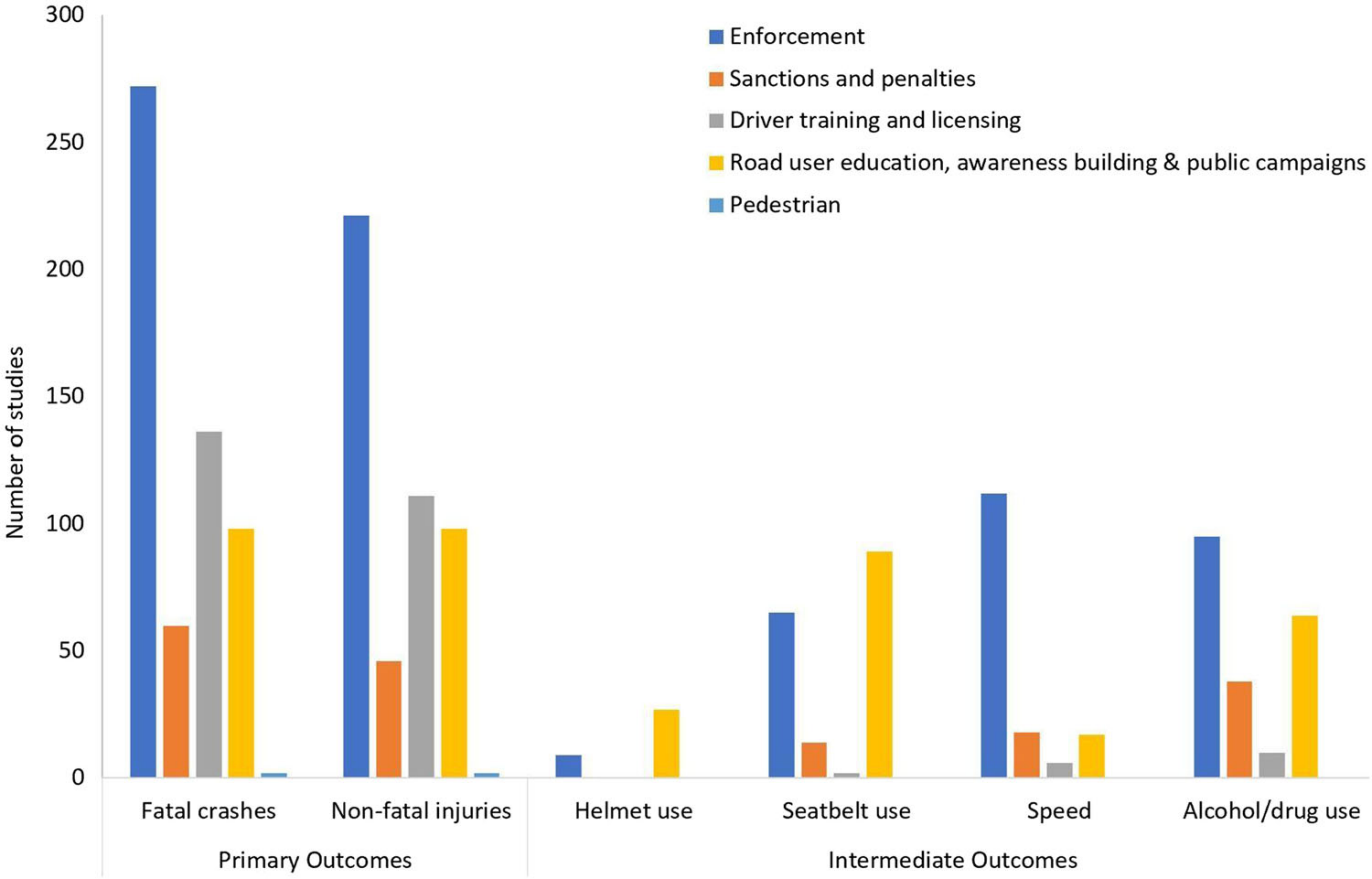
Human Factors Interventions: Number of studies by type of outcome.

We identified a total of 771 records that focused on interventions related to human factors (Figure 7). Of these, 651 were impact evaluations and 120 were systematic reviews or protocols. In terms of types of interventions, about half of the studies reported interventions of enforcement (*n* = 401), about one‐third reported evaluation of road user education (232), one‐fifth reported driver training and licensing (146), and the rest included sanctions and penalties (99) or pedestrian (2). Note that some studies are categorized under more than one intervention.

The map further classified these studies based on the four behavioral or intermediate outcomes. Studies with alcohol use as an outcome were most common (207), followed by an equal share of seatbelt use (138) and speed (142), and the studies for helmet use were least common (33). Next we look at the types of interventions under which different outcomes were studied. Helmet use as an outcome has been studied mostly with the interventions of road user education (70%) and the rest with enforcement. In the case of speed as an outcome measure, it is the reverse. It has been studied mostly with enforcement (76%) and the rest with sanctions (10%) and road user education (11%). Seat‐belt use has been studied about equally with enforcement (40%) and road user education (51%). Alcohol use has been studied most often with enforcement (47%), followed by road user education (29%).

Among the systematic reviews, those for road user education were the most common (35%), followed by an almost equal share of driver training and license (25%) and enforcement (29%). Sanction and penalties (10%) and pedestrians (1%) had the least number of systematic reviews.

Ninety‐nine percent of these studies were conducted in either high‐income countries (95%) or upper‐middle income countries (4%). Among the world regions, 96% of the studies were conducted in North America (59%), East Asia and Pacific (19%) and Europe and Central Asia (18%). The distributions of studies into different world regions did not vary significantly across different interventions. More than three‐fourths of all driver training studies were conducted in North America, highest across all interventions, while only 6% of those were from Europe and Central Asia. Europe and Central Asia had their greatest share in studies of sanctions and penalties (26%).

Among the impact evaluations, about 60% of the studies used a before‐after approach, another quarter used time‐series analysis and the rest used case‐control or experimental studies. This distribution of study design varied over different types of interventions. Among road user education and enforcement studies, before‐after studies were most common, with a share of 67% and 62%, respectively. Among sanctions and penalties, the most common method was time‐series (42%) followed by before‐after (38%). Experiments were most commonly used in road user education evaluations (15%), while in other interventions, these study methods were highly uncommon (2%–6%). Case‐control studies were twice as common in sanctions and driver training (10%–12%) than in enforcement and road user education (5%–6%)

### 6.5 Vehicle factors & protective devices

**Figure 8:**
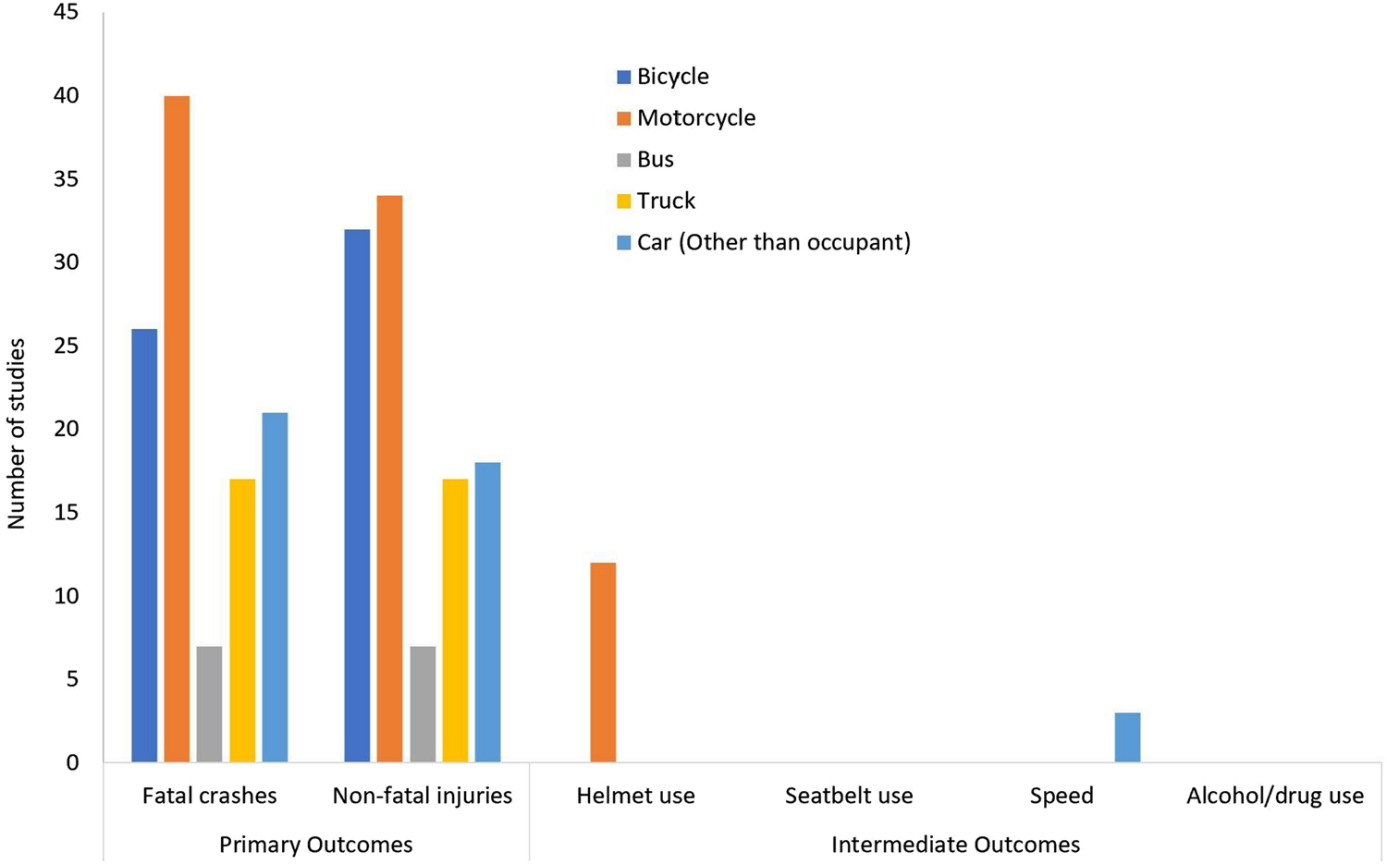
Vehicle Design Interventions: Number of studies by type of outcome.

The map identified 106 studies (65 impact evaluations and 41 systematic reviews) that focused on interventions related to Vehicle Factors and Protective Devices (Figure 8). Among these, motorcycles were the focus of most studies (46), followed by bicycles (33), Car: non‐occupants (21), and trucks (18). There were very few studies that focused on buses (7) and none that focused on the non‐specific category of Other Vehicles. In most vehicle domains, studies were relatively equally split between evaluating fatal and non‐fatal outcomes, with many studies that included both. Almost all studies (85%) were from high‐income countries. There was only one impact evaluation from a low‐income country, and only one from a lower‐middle income country (Cambodia). Most studies were primarily from the regions of North America, Europe and Central Asia, and East Asia and Pacific (which includes Australia, New Zealand, and Japan). There were no impact evaluations from Latin America, the Caribbean, the Middle East or North Africa. Case‐control studies were the most common study design and were used often for the evaluation of motorcycle and bicycle helmet effectiveness. None of the studies used an experimental study design.

Among the 33 impact evaluations and 13 systematic reviews that were related to motorcycles, more than two‐thirds (32 studies) focused on the effectiveness of helmets in preventing motorcyclist injuries. In addition, 4 studies assessed the effects of improved braking systems (including anti‐lock brakes), 3 assessed the use of daytime running lights, 2 were on the effects of motorcycle engine capacity, and 3 were on protective clothing, use of reflectors and other means to improve conspicuity.

Among the 23 impact evaluations and 10 systematic reviews that were related to bicycles, almost all studies (28) were related to the effectiveness of helmets in preventing injuries to bicyclists. Of the remaining 5 studies, most (4) focused on interventions to enhance the visibility of bicyclists through reflectors or clothing.

Out of the 24 studies on how car design factors affect non‐occupants, only 10 were impact evaluations. The technologies evaluated included daytime running lights, antilock brakes, front‐end shape, intelligent speed adaption, speed limiters, and a package of safety regulations. The remaining 14 were systematic reviews and included evaluations of braking systems (including antilock brakes, electronic stability control, automated braking and brake assist technologies), blind spot detection, bull bars, improved headlights (including daytime running lights), vehicle shape and front‐end design, and vehicle reversing cameras.

Out of the 18 studies related to trucks, only 7 were impact evaluations. The technologies and interventions evaluated included crash attenuators mounted on the back of trucks, speed limiters, ISO‐9000 certification, conspicuity treatments, daytime running lights, and motor carrier safety audits. The remaining 11 systematic reviews focused on braking systems (including antilock brakes and automatic emergency braking), blind spot detection, bull bars, vehicle inspections, safety equipment on vehicles, safety standards for trailers, and under‐run guards.

Out of the 7 studies related to buses, there were 3 impact evaluations (one each on speed limiters, daytime running lights and motor carrier safety audits. The 4 systematic reviews focused on periodic inspection, safety equipment, and bull bars.

### 6.6 Road design infrastructure and traffic control

**Figure 9:**
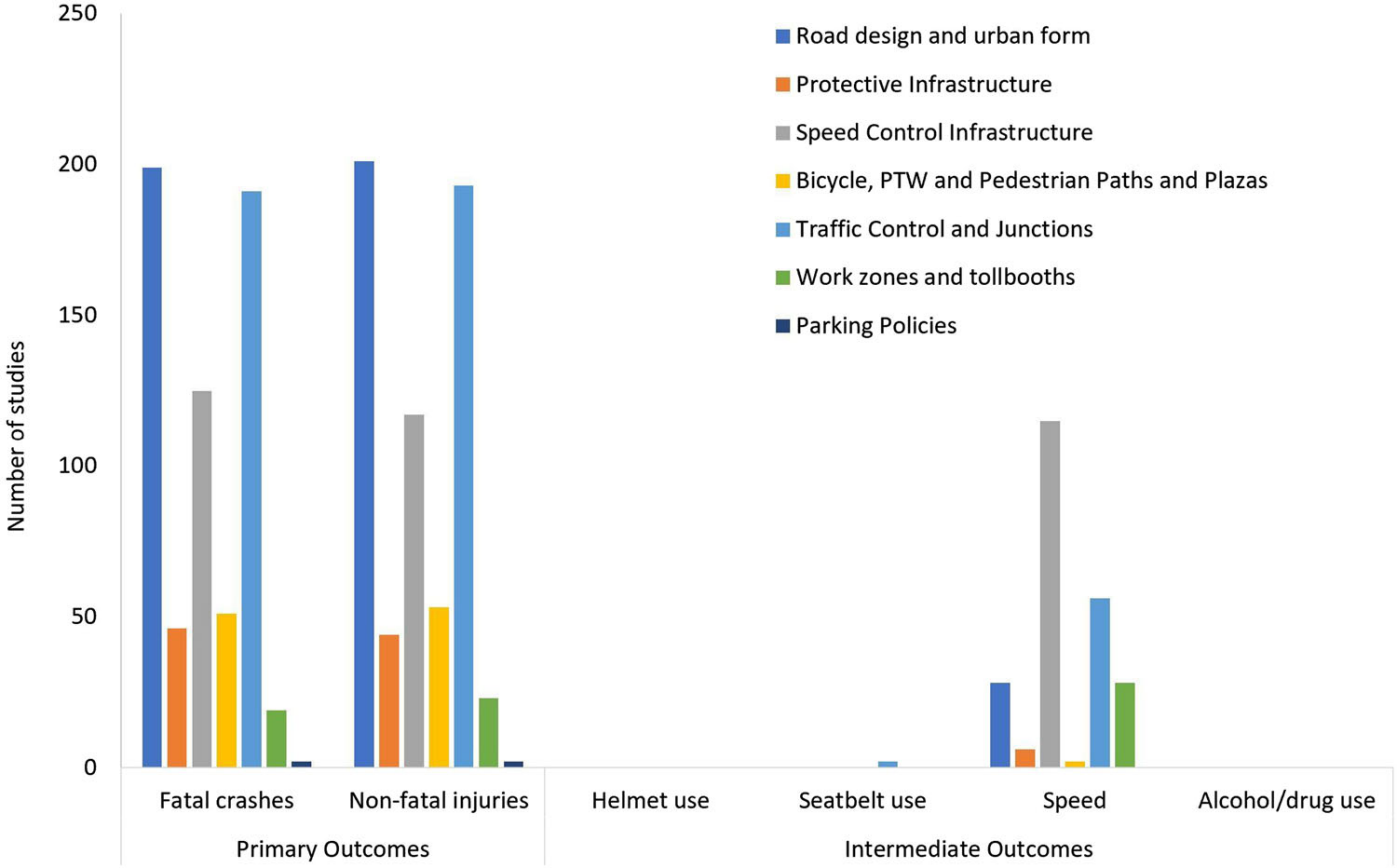
Road Design and Infrastructure Interventions: Number of studies by type of outcome.

There are 7 intervention categories under road design infrastructure and traffic control. The largest number of studies are available for traffic control and junctions with 192 impact evaluations and 47 systematic reviews (Figure 9). This is followed by the two intervention categories of road design and urban form (159 impact evaluations and 58 systematic reviews) and speed control infrastructure (171 impact evaluations and 16 systematic reviews). The other three categories that have much lower number of studies are bicycle, MTW and pedestrian paths and plazas, work zones and toll booths, and protective infrastructure. The intervention category of parking policies has only one impact evaluation and systematic review each.

Most of the systematic reviews were of low quality. Only one high‐quality systematic review is available for road design and urban form and one for speed control measures. Most impact evaluation studies have followed before‐after study design, with 75% of impact evaluations using this method. Case‐control and time‐series were used for 10% and 6% of studies, respectively. Case‐control was most used for studies related to bicycle, MTW and pedestrian paths with one‐fifth of these studies using this method. Time‐series was most used for speed control infrastructure with one‐tenth of the studies using this method.

Overall, there were twice as many studies reporting outcomes for motorised modes than for non‐motorised modes. This ratio was most divergent in the case of protective features where motorised modes were more than five times more likely than non‐motorised modes. In bicycle, MTW, and pedestrian paths, as expected, the ratio was reversed, and non‐motorised modes were about three times as likely as motorised modes.

Almost all the studies (96%) have been carried out in high‐income countries. Among different geographies, North America contributed 54% of the studies, Europe and Central Asia, another 26%, and East Asia and Pacific, 16%. There were almost no studies from South Asia, Sub‐Saharan Africa, orLatin America and the Caribbean.

There were more than twice as many studies that reported outcomes in urban areas than in rural areas. Within each of the interventions, there were variations in the urban‐rural distribution. Road design and speed control were equally reported in urban and rural areas. Almost all the studies for bicycle, MTW and pedestrian paths have been conducted in urban areas. Traffic control and junctions were twice as likely to be reported from urban areas than rural areas.

### 6.7 Post‐crash pre‐hospital care

**Figure 10:**
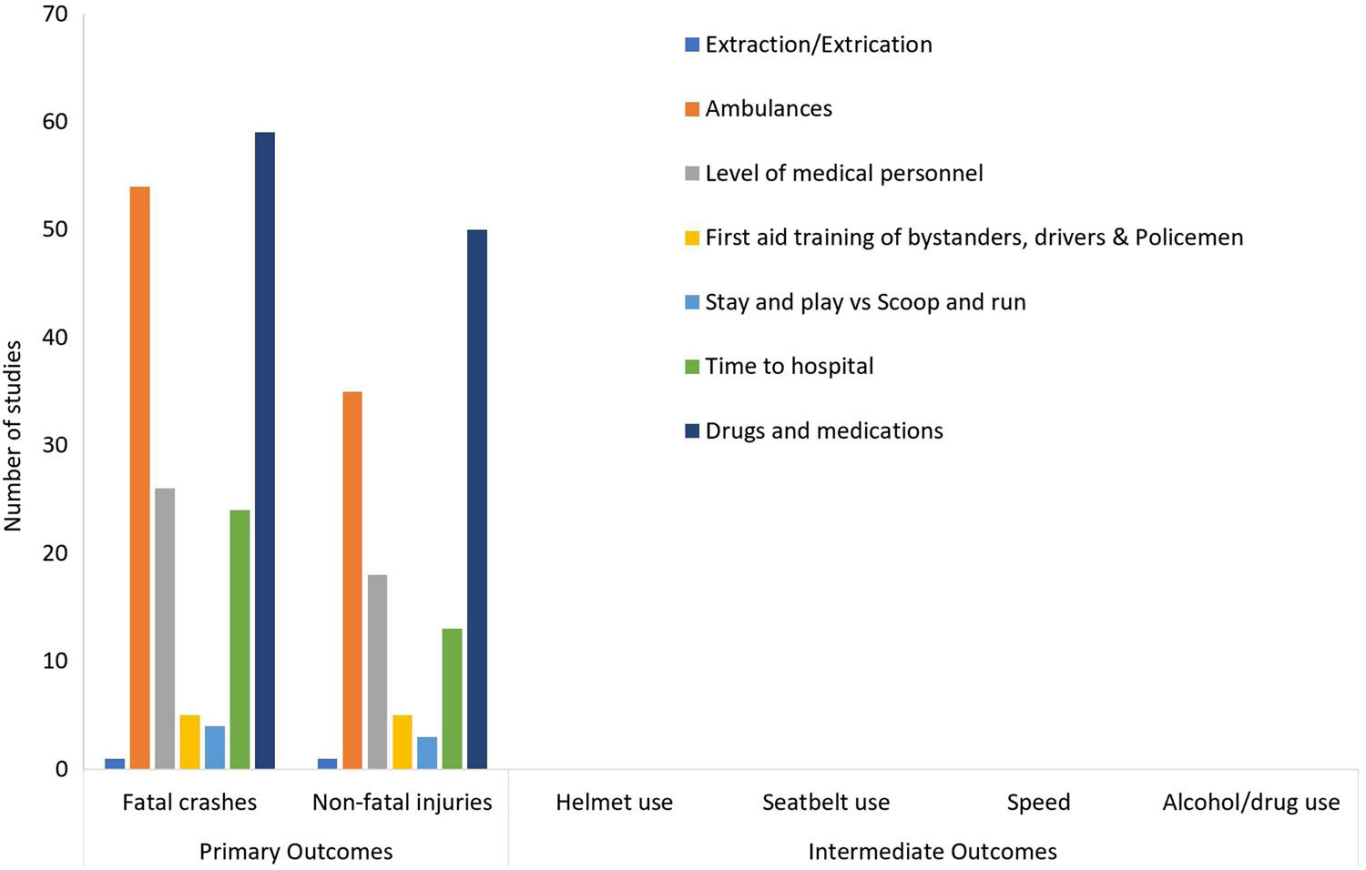
Post‐crash Care Interventions: Number of studies by type of outcome.

We identified a total of 118 studies (27 systematic reviews, 86 impact evaluations and 5 protocols) (Figure 10). Nearly two‐thirds of the studies focused on ambulances (10 systematic reviews and 47 impact evaluations) or drugs and medications (14 systematic reviews, 40 impact evaluations and 5 protocols). The two intervention categories of level of medical personnel (27) and time to hospital (24) comprised 29% of the studies. Finally, the remaining intervention categories such as first aid training (5), stay and play vs scoop and run (4) and extraction/extrication (1) were few in number.

In all the studies the outcomes were a reduction in post‐crash fatalities and some of them also indicated reduction in non‐fatal injuries.

Nearly 80% of the studies were from the USA, Australia, Japan and major European countries. Higher middle‐income countries such as China, Brazil, etc. accounted for 12% of the studies. Low and lower middle‐income countries including those from South Asia and the African continent comprised of the remainder 8% of the studies. A large proportion (42%) of the studies were reported from the USA and Canada. This was followed by the European and Central Asian countries (28%) like the UK, France, and Iran. Next, the East Asian and Pacific countries like Australia, Japan and China contributed 16% of the studies. Among the regions with the lowest shares, the African countries contributed 7% and South Asian and Latin American countries contributed 6% of the studies.

Nearly all the studies evaluated the effect of trauma which involved victims from all types travel modes (cars, MTW, pedestrians and bicyclists). Amongst these 78% of the studies focused on the treatment of adult population. Unless specifically mentioned, patient groups such as pregnant women and patients with co‐morbidities were excluded. There were 22% of the studies that focused on post‐crash trauma care of children and teenagers.

Among the methodologies used, 74% of the studies were randomised control trials, case control or systematic reviews. Most of these study designs have been quite common in medical and public health research, but this is quite different from what we see in general road safety literature where before‐after and time‐series based studies are more common. RCTs were most commonly used in the interventions of drugs and medication, while in the evaluations of ambulances, case‐control was the most common and RCTs were second most common.

### 6.8 Legal and institutional framework

**Figure 11:**
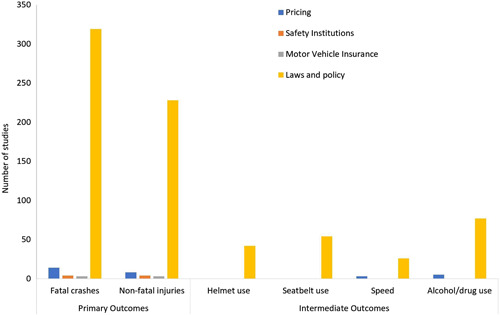
Legal and Institutional Interventions: Number of studies by type of outcome.

We identified a total of 424 studies that focused on interventions related to legislation (Figure 11). Within these, there are 35 systematic reviews and 389 impact evaluations. Almost all the studies were on laws and policy (406), and only a few on pricing (20), safety institutions (4), and motor vehicle insurance (4). All the interventions reported fatal or injury crashes as the outcomes. However, only the studies on law and policy and those on pricing reported one of the four intermediate outcomes of change in speed, change in alcohol use, seat‐belt and helmet use.

Among the studies on laws and policy, change in alcohol use was most commonly reported (*n* = 71), followed by seatbelt use (*n* = 51), helmet use (36), and change in speed (28). Many of these studies included evaluation of interventions such as a new legislation decreasing permissible blood alcohol level (Nagata et al. 2008), implementation of speed limits (e.g., Bornioli et al., 2018), law mandating compulsory use of seat belts (e.g., Foldvary & Jane, 1974) or law mandating use of motorcycle helmets (Olsen et al., 2016).

Almost all the studies (95%) were based in high‐income countries. There were only 4 impact evaluations and 2 systematic reviews from low‐and‐middle and low‐income countries, all with a focus on laws and policy. The overrepresentation of high‐income countries is reflected in the regional distribution of the studies. North America alone comprises two‐thirds of all the studies, and Europe and Central Asia, and East Asia and Pacific, comprising 14% each. Before‐after and time series are the two most common study designs, each comprising 45% of the studies.

## 7 DISCUSSION

### 7.1 Summary of main results

As indicated by the distribution of studies across world regions, among all the impact evaluations and systematic reviews included in this EGM, less than 10% were conducted in LMICs. As a result, the types of interventions that have been studied more frequently are biased towards road safety needs of high‐income countries with high levels of car use. Much of the historical reduction in road deaths in these countries starting in the 1970s was achieved through interventions focused on car occupants such as car design or seatbelts. Indeed, it is the accumulation of this research over past 50 years that dominates road safety literature today. However, in LMICs, for example in many countries of Latin America, sub‐Saharan Africa and Asia, there is a lot more heterogeneity in traffic. Road users outside the cars such as pedestrians, cyclists, and motorcyclists are often in the majority, and they share road space with cars, buses, and trucks. Due to the underrepresentation of LMICs in scientific literature, there is a general lack of evidence for interventions that may have greater applicability in these countries.

For example, even though motorcyclists form a significant share of road deaths in many LMICs, there are only a few studies that have focused on interventions related to the safety of motorcyclists. These include studies evaluating new technologies in motorcycles (e.g., ABS), the design of helmet to suit tropical climates, or interventions to improve the prevalence of helmet use. In the same manner, even though trucks and buses contribute to a large share of pedestrian deaths in LMICs, studies that evaluated the effectiveness of improved front‐end design for buses and trucks to reduce injury severity of impacting pedestrians are lacking. In some instances, while a particular intervention may have been evaluated in a high‐income country context, it may not be directly applicable in other contexts. For example, traffic calming techniques (e.g., roundabouts) have been implemented extensively in many urban areas of high‐income countries and have been shown to be highly effective. However, the effectiveness of traffic calming in reducing the speed of motorcycles is not known and therefore more evidence may be needed from LMICs with high levels of motorcycle use. The EGM also points to lack of good‐quality systematic reviews. In other words, the available evidence of the interventions that have been studied also needs greater scrutiny or more research.

### 7.2 Areas of major gaps in the evidence

#### 7.2.1 Human factors

Among the interventions categorized under human factors, there was a paucity in the studies that evaluated helmet use as an outcome. This is a direct implication of the regional representation in the studies. Since, almost 95% of these studies were conducted in high‐income countries, where motorcycle usage is low or rare, helmet studies have not been of priority. More than 90% of all road deaths are contributed by LMICs and in many of these countries, motorcyclists contribute up to 40% of all road deaths (WHO, 2018). In South Asia, for example, the region with one of the highest levels of motorcycle use, the map reported only 3 impact evaluation studies related to helmet use. Future research agenda in LMICs should urgently address this gap.

The map also shows that helmet prevalence was more than twice as likely to be studied in interventions of road user education than in interventions of enforcement. Further, there is only one systematic review of the impact of enforcement on helmet use, while there are 7 systematic reviews of the impact of road user education on helmet use. This shows a likely bias towards safety education efforts both in practice as well as in research. Seat belt use is another outcome measure where road user education was the intervention for half of all studies. However, this large bias towards road user education is not seen in speed and alcohol as outcomes. For example, for speed, only 10% of the studies were based on educational interventions, and in alcohol use, 30%.

Given that almost all studies were conducted in high‐income countries, it is important to assess the evidence for its transferability to LMIC settings. The vehicular mix in high‐income countries is dominated by cars, while in LMICs, it is a heterogenous mix of cars, motorcycles, cycles, buses, and various types of para‐transit modes. Thus a ‘driver’ is not just a car occupant but represents users of a variety of transport modes. With such variety, measures like enforcement, road user education, and driver training, will have to be calibrated according to different road users.

#### 7.2.2 Vehicle factors and protective devices

Among the interventions categorized as Vehicle Factors and Protective Devices, we excluded car technologies that improve occupant safety from this EGM because there is a large existing literature that has systematically reviewed this topic. Nevertheless, it is important to note here that improvement in car safety technologies have had a particularly profound impact on improving occupant safety in high‐income countries. In fact, a review by the US National Highway Traffic Safety Administration concluded that vehicle design improvements and increasing belt use in the US between 1960 and 2012 reduced traffic mortality by as much as all other factors combined (Kahane, 2015), and the widespread use of these proven technologies in LMICs will provide large safety gains (Bhalla and Gleason 2020). This success with car‐related technologies is in sharp contrast to the large gaps in evidence for technologies focused on other types of vehicles and on protecting other types of road users (i.e., non car occupants).

There are many car technologies that can reduce risk to pedestrians, bicyclists, and motorcyclists. Among these, there are multiple evaluations, including high‐quality systematic reviews focused on the effects of electronic stability control (Elvik, 2009; Hoye, 2011). However, there is a dearth of evaluations on other potentially important technologies, such as vehicle front‐end design, automatic braking, and improved headlights. Although, European directives started requiring improvement of the front‐end design of cars for pedestrian protection starting in 2005, only one evaluation of the resulting changes in car design met the EGM's inclusion criteria. This is important because improved front‐end design is one of the 8 priority regulations promoted by the UN World Forum for Harmonization of Vehicle Regulations for adoption by LMICs. Notably these regulations only apply to cars and exclude buses and trucks, which are responsible for most of the pedestrian impacts in some countries (Bhalla & Gleason, 2020). While there have been several studies focused on developing bus‐ and truck‐front designs (Kajzer et al., 1992; Chawla et al., 1999; Feist et al., 2008), we found no real‐world evaluations of such interventions.

The literature evaluating motorcycle‐related technologies is not commensurate with the scale of the problem. Motorcyclists are the most common road users killed in traffic crashes in many LMICs specially in South Asia, East Asia and Pacific, and Latin America and Caribbean (James et al., 2020). Studies evaluating the design of motorcycle helmets are a notable exception with many evaluations and systematic reviews. However, almost all of these studies evaluate the ability of helmets to attenuate crash forces. Although these studies have primarily been conducted in high‐income countries, helmet designs are essentially the same worldwide (polystyrene foam inside a polycarbonate shell) and the biomechanical tolerance to head injury doesn't vary substantially across populations in different countries. Therefore, further similar evaluations in more countries are unlikely to yield new insights. However, there is a pressing need for evaluations of helmet designs that encourage helmet use, such as by providing better ventilation (Patel & Mohan, 2003) and encouraging correct use, such as by proper fixation (Ramli & Oxley, 2016). As with motorcycles, the bulk of the bicycle‐related studies identified were focused on helmets, particularly on the ability of helmets to reduce injuries when worn correctly. There is a need for further studies that focus on helmet characteristics that can increase proper use in diverse environments.

Beyond helmets, there were few studies on other motorcycle‐related technologies. We identified three evaluations of motorcycle anti‐lock brakes that report relatively large reductions in RTI. However, even a decade after the first of these studies, there are no evaluations that assess ABS effectiveness in small‐engine capacity (<125cc) motorcycles that comprise the majority MTWs in most LMICs. There are also significant gaps in the evaluation of other motorcycle‐related technologies (such as leg‐guards, improved head lamps, protective clothing, and daytime running lights).

Most of the studies come from high‐income countries in North America, Europe, and Central Asia, and East Asia and Pacific. The implication is that there are no evaluations of issues that are uncommon in high‐income countries but are important across many LMIC settings. For instance, there is a wide range of indigenous vehicles, such as autorickshaws, tuk‐tuks, tempos, and jeepneys, that are used extensively in LMICs. Some of these vehicles have been the focus of engineering design studies to develop countermeasures including removal of sharp objects, use of padded surfaces, improvements in rollover characteristics, modifications to reduce intrusion and improve structural crashworthiness, and means to restrict passenger ejection (Mohan et al., 1997; Chawla et al., 2001; Mukherjee et al., 2007; Gawade et al., 2004). Nevertheless, we found no studies that have evaluated the effects of such interventions in real‐world crashes. There is a pressing need for studies that assess interventions that address the most important vehicle‐design issues in LMICs, where the bulk of RTI and deaths happen.

#### 7.2.3 Road design infrastructure and traffic control

There are two major limitations in the evidence available for road design interventions. First, almost all the studies have been carried out in HICs, with the majority originating from North America. The traffic mix where these studies have been carried out is different from the traffic existing in LMICs, especially in South Asia and Sub‐Saharan Africa. Thus the available evidence may not translate to LMICs. Second, most systematic reviews are of poor quality. This means that the available evidence synthesis may either need greater scrutiny or more empirical research.

Traffic crash patterns in LMICs can be substantially different from those in North America and Western Europe. In the US, for example, pedestrians, cyclists and motorcyclists comprise 30% of all road deaths (WHO, 2018). In comparison, in sub‐Saharan Africa, this share is more than 50% (Damsere‐Derry et al., 2017; Adeloye et al., 2016; Solagberu et al., 2015) and in India, this is more than 70%. In India, even on rural highways, pedestrian and motorcyclists have a greater share among road deaths than other road users (Mohan et al., 2017). These crash patterns are somewhat similar to those observed in urban areas. On the other hand, the involvement of pedestrians, cyclists and MTW users on rural highways in a high‐income country would be a rare occurrence.

Given this contrast, the evidence from a high‐income country context may or may not be applicable to LMICs depending on the intervention. For example, the results involving the effectiveness of crash barriers for cars and heavy vehicles may be broadly valid for all geographical regions. However the use and safety performance of design features such as wide shoulders may be different in LMICs due to the presence of slow‐moving vehicles on high‐speed roads that may use shoulder space as a travel lane. Upgradation of highways involving improved pavement surface, wider lanes, and wider shoulders may lead to higher speeds and more frequent lane changing and conflicts. Pedestrians and slow vehicles on the curb‐side lane or shoulders will be exposed to motorised vehicles moving at much higher speeds. Therefore, highway design standards applicable to highways in high‐income countries may not have the same level of effectiveness in ensuring the safe movement of traffic in LMICs, and the safety benefits of road upgradation using present design standards remains unclear for LMICs.

Similarly, in urban areas, traffic calming techniques that use road design changes to reduce the speed of vehicles often include narrowing road widths, adding curvature to a straight road section, replacing intersections with roundabouts, or using smaller radii of curvature at the turns. Many of these interventions may work for cars but may not be equally effective for motorcycles that may negotiate through these design changes without much deflection.

Most studies have focussed on motorised traffic. This is understandable given the importance of motorised traffic in high‐income countries. However, in view of the current focus on SDGs, non‐motorised traffic and active transport modes have acquired importance in both HICs as well as other countries (Costanza et al., 2016). Therefore future efforts are required for evaluating the impacts on these road users.

New research is required to make cities safer for pedestrians in all countries, such as revisiting current speed limits, lowering speed limits, and also active measures (such as speed bumps) to control speeds. There is consensus on urban design principles that can lead to safer cities (higher mixed land‐use density) – the conditions that are already present in less‐motorised countries. There is a need to relook at city planning paradigms moving from a car‐friendly to a people‐friendly city. New research is required to get insights from these settings and an understanding of barriers to the implementation of these principles.

LMICs and low‐income countries have to make stronger efforts to understand the requirement of pedestrians along high‐speed roads and develop new standards for ensuring pedestrian safety along these facilities.

#### 7.2.4 Post‐crash pre‐hospital care

There are two major paradigms of post‐crash pre‐hospital patient care: (1) Stay and Play, which focuses on extrication, onsite stabilization of the patient, and transportation of the patient (with enroute interventions to continue stabilisation) to a definitive care facility; and (2) Scoop and Run, which aims to extricate and transport the patient to a medical facility for further care as soon as possible. Extrication is not considered significantly important by many clinicians; however, it can influence the outcome. Therefore, the lack of significant literature on this issue is a matter of concern. The intervention categories in this section show high overlap with each other.

A large proportion of the studies have focused on ambulances, and many of these were conducted in HICs. However, in LMICs, ambulances are not commonly used for the transport of crash victims to the hospital (Bhalla et al., 2019). Instead, most victims are transported by passing vehicles or, in some settings, by traffic police. Studies evaluating what improves outcomes after layperson transport are urgently needed. Only 18 out of 130 impact evaluations looked at time to hospital and the effect of delayed care, and most of those were conducted in HICs. Existing evidence suggests that a survival benefit exists in patients arriving earlier at hospital after severe head injury, but the benefit may extend beyond the golden hour (Dinh et al., 2013). However, the extent to which such findings apply to LMICs is unclear because of vast differences in the ability of hospitals to provide life‐saving care. Similarly, consider that available evidence suggests that first‐aid training improves first‐aid knowledge, skills, and self‐confidence immediately after training and for 3 months or longer after training. However, research on the possible effects of first‐aid training on traffic mortality is scarce (Martensen et al., 2019). Therefore, merely improving prehospital care without improving definitive care capacity in the hospital may only shift the mortality statistics from the roadside to the hospital. These issues can only be properly addressed by conducting evaluations of interventions in LMIC settings.

Regarding the level of training of EMS personnel, the EGM found few impact evaluations. In a systematic review Jayaraman et al. (2014) concluded that accounting for the trauma score of the patient, providing specialised training to EMS personnel did not result in significant improvements in outcomes. But in each of these cases, the type of trauma is a significant variable in deciding the survival rate of the patient. As an example, in the case of penetrating injuries, basic life support (BLS) seems to be the proper care treatment, whereas in blunt head injuries, advanced life support systems show better patient recovery (Ryynänen, 2010). This is an intervention that can be replicated in other LMICs and LICs in theory and is something that is being attempted. Although one also has to consider the economic impact of upgrading not only the staff but also the equipment used in the ambulances. And unless the number of such specialized ambulance units grow substantially benefits might not be significantly visible. Furthermore, the review found, based on the limited data, that there is at present no evidence to recommend ALS training of ambulance crews to care for injury victims. Also, definitive care capacity at the receiving hospital will affect the outcome of trauma (Jayaraman et al., 2014).

Notably, the EGM shows that medicinal interventions in the form of drugs and medicines administered to patients in transit have been studied in many studies. Bleeding control and management of the patient is the most important part of this, for which the use of tranexamic acid showed the best results in multiple studies including the CRASH 2 trials in 2011 conducted in multiple countries. Intravenous fluid replacement to offset volume of blood loss is an intervention frequently recommended in all emergency and prehospital care of trauma victims. In the case of intravenous fluid replacement either by colloidal or crystalloid solutions, Lewis (2018) noted no significant difference between the two. Kwan et al. (2014) in a Cochrane review found no evidence to either support or not support the use of early or larger volume intravenous fluid in uncontrolled bleeding.

Even though the speed of transport (within the ‘golden hour’) is discussed extensively in road safety policy documents, there are relatively few robust studies that have evaluated the effect of transfer time on outcomes. Sirens and flashing lights and faster‐moving ambulances have been emphasised for this and they have become the norm to define the identity of ambulance movement. Although only 75% of the ambulances are run with warning lights and sirens (WLS), a disproportionate 91% of response mode collisions were during a WLS response (Custalow et al., 2004). To further emphasise the speed of transportation there has been much discussion on the effectiveness of helicopter or air patient transport versus conventional road transport. Despite the high expense of such transport, there are few robust evaluations of their outcomes. And, the results of the studies are mixed, with only some reporting benefits. Studies by Zigmond (2008) and Snooks et al. (1996) noted that helicopter EMS (HEMS) did not result in any appreciable change in outcome. Most importantly, helicopter‐based studies are concentrated mostly in HICs. This is expected due to the higher cost of maintaining a HEMS fleet because of which majority of these studies are conducted in countries in North America and Europe. The replicability of HEMS in LICs and LMICs therefore remains uncertain.

No systematic review or controlled studies were found for the following: scoop stretchers, spinal immobilization boards, head stabilizers, cervical collars, intubation equipment, artificial breathing apparatus, and mechanical CPR machine.

#### 7.2.5 Legal and institutional framework

Legislations form the foundation needed for the successful implementation of traffic enforcement programs. As many LMICs continue to witness growth in traffic and RTI, an important intervention will be the passing of legislation that addresses road safety issues of the country. However, we found few studies from LMICs that evaluated the impact of legislative interventions. This is expected partly due to the paucity of road safety legislation in many parts of the world. WHO conducted a worldwide review of legislation for the five risk factors – speeding, drink‐driving, use of motorcycle helmets, use of seatbelts, and use of child restraints. Among the 175 countries reviewed, only 25 of those had laws meeting best practices for 4 or 5 of those risk factors. Many without legislation tend to be LMICs. For example, a large proportion of the countries with no legislation for 50 km/h speed limit on urban roads are LMICs (WHO, 2018).

The transferability of the evidence of legislative measures from HICs to LMICs is particularly problematic because the effectiveness of a law to achieve on‐road compliance is highly dependent on enforcement and policing. In other words, the relationship between legislation and its impact on traffic safety is mediated by enforcement. Studies evaluating legislation often do not report the level of enforcement. This is because many of these interventions involve a new legislation, which may simply be enforced by the police as an additional traffic offense without changing the intensity or coverage of enforcement. Consequently, evaluations of legislation may not have external validity.

The literature on road safety institutions is scarce. This is noteworthy given the importance of such institutions to improve safety in countries. According to international experience, it is almost impossible to promote safety in a comprehensive and scientific manner without an independent road traffic safety agency (Mohan et al., 2017). However, the scarcity is expected because interventions of setting up new institutions cannot be frequent. An institution once established in a country continue to function for a long time. National Highway Traffic Safety Administration (NHTSA) in USA was established in 1970 under the U.S. Department of Transportation, was established by the Highway Safety Act of 1970, as the successor to the National Highway Safety Bureau, to carry out safety programs under the National Traffic and Motor Vehicle Safety Act of 1966 and the Highway Safety Act of 1966. NHTSA continues to improve traffic safety by setting and enforcing safety performance standards for motor vehicles and motor vehicle equipment, and through grants to state and local governments to enable them to conduct effective local highway safety programs. There are also methodological challenges in evaluating the impact of institutions. Just as legislation works through its enforcement, institutions do not have a direct impact on safety. They improve road safety through the formulation of multiple interventions, and it's the latter that are evaluated in road safety studies. The interventions of pricing are often based on natural experiments unrelated to traffic safety or not solely related to it. These include changes in fuel prices, taxation on alcohol, and congestion pricing. Interestingly, even with widespread use of motor vehicle insurance across the world, there are only three impact evaluations.

### 7.3 Limitations

The EGM focuses on the availability of evidence for interventions but does not provide a synthesis on the effect of these interventions on outcomes, as is typically done in systematic reviews. In fact, beyond effectiveness, the interventions reviewed in the EGM are disparate representing the range of opportunities available to policymakers to improve road safety. These interventions differ on a range of dimensions, including their cost, their targets (humans, infrastructure, vehicles, etc.), and the timeline over which interventions are conducted and benefits accrue. Consider for instance that the effects of most enforcement and social marketing campaigns are transient while the effects of infrastructure are long‐lasting, such that small short‐term benefits can accrue over time (Bjornskau & Elvik, 1992). Such distinctions are important considerations in policy debates but are beyond the scope of an EGM. Similarly, safety projects typically involve multiple interventions. While the EGM database is coded to include all interventions that were included in the study (the map allows studies to be filtered [with AND/OR operators] to any combination of interventions), the analysis presented here focuses on the individual interventions. Assessing the relative role of multiple interventions is beyond the scope of the EGM and requires systematic reviews. Finally, note that the lack of evidence is not the only reason why proven interventions are not implemented in road safety – that is, there is an implementation gap. Although the purpose of this EGM is to focus on the research gap (i.e., do we know what works and what doesn't?), we also need to understand how to get what is known to work implemented.

Understanding issues of diversity and equity are important considerations in understanding the impacts of road safety interventions and policies on different populations. The EGM allows exploring some of these issues directly (e.g., by assessing evidence gaps in LMICs relative to HICs) and others indirectly (e.g., assessing evidence for safety of pedestrians, who are often poorer than occupants). However, a proper exploration of equity impacts would require a systematic effort to define variables that characterize equity and extract the availability of this information from the road safety literature. Although such analysis was beyond the scope of this EGM, such studies should be undertaken using this EGM as a starting point.

## 8 AUTHORS’ CONCLUSIONS

### 8.1 Implications of findings

This EGM illustrates that while there is a large literature that empirically evaluates what interventions work (and what don't work) in road safety, these studies are very unevenly distributed globally. The vast majority of the studies have been conducted in the high‐income countries in North America, Western Europe and East Asia and Pacific. This is an issue because the external validity of a study (i.e., the extent to which research findings in a population can be generalized to other settings) depends on the type of intervention. For instance, studies that assess the effectiveness of crashworthiness technologies (e.g., effectiveness of helmets, seatbelts, airbags, etc.) have high external validity because the injury tolerance of the human body is reasonably similar across countries. Primarily for this reason, we excluded vehicle design technologies for occupant protection from this EGM (there is a large literature evaluating these technologies; and the findings of these studies are valid in other settings as well).

However, in other domains (e.g., road design, human factors, legal and institutional factors), contextual factors can dominate. For instance, consider that the traffic environment on rural roads and highways in India is substantively different from that in the US. While rural roads are primarily used by cars in the US (and most crash victims are car occupants), in India, rural roads will often have much more heterogenous traffic, with a significant presence of pedestrians, bicyclists, motorcyclists, and autorickshaws. Consequently, interventions that are protective in the US (e.g., paving of shoulders, which reduces crashes by providing straying vehicles room to recover) could lead to an increase in crashes in India (e.g., paved shoulders lead to increased speeds that are lethal to pedestrians and other vulnerable road users). Nevertheless, nearly all of the research on the design of safer roads has been conducted in the US, Australia, New Zealand, and Western Europe. In the absence of local research, LMICs (many of which are rapidly expanding their road infrastructure) are adopting design standards and guidelines developed based on research conducted in HICs. This situation can only be remedied by increasing the amount of research undertaken in LMICs.

Beyond the problem of external validity of interventions that have been evaluated, the EGM illustrates that there are many important road safety problems that have not received attention, primarily because they do not occur in HICs. For example, this includes addressing motorcycle‐pedestrian crashes, which are common in countries where motorcycles are common. Similarly, although vehicle technologies for occupant protection have been extensively evaluated, there are no evaluations that focus on autorickshaws, tuk‐tuks, and other vehicles that are primarily produced and used in LMICs. Likewise, we found no evaluations that addressed the safety of unrestrained occupants in the backs of trucks and agricultural tractors, which is a common occurrence in many LMICs. And, as a final example, the EGM shows that the post‐crash care literature focuses almost exclusively on evaluating ambulances. However, most victims of traffic crashes in LMICs are transported by taxis and other passing vehicles. We found few interventions that aimed to improve the outcomes of layperson transport. Again, there is an urgent need to fill these gaps in evidence through well‐designed research studies.

Simultaneous with the need to increase research in LMICs, is the need to increase the use of high‐quality research methods. In recent years, the research output from many LMICs has dramatically increased. Our initial search for evaluations identified many papers from China, India, Iran, Brazil, and other LMICs on road safety applications, especially those related to transportation engineering. In fact, many of these countries have a large pool of well‐trained transportation engineers. However, most of these studies did not meet our inclusion criteria, most often because they used cross‐sectional study designs with multivariate regression, which is often poorly suited for causal inference. In biomedical research, randomized experiments are considered the highest form of evidence, but such studies are usually not logistically feasible for most road safety interventions. This is reflected in the small number of experiments that were identified in this EGM. Nevertheless, the EGM shows that in high‐income countries, the use of observational epidemiological approaches (e.g., the case‐control method) has been deployed extensively to control for many of the sources of variation in cross‐sectional data. Similarly, well‐designed observational before‐after studies can have high validity but are rarely used in LMICs. Therefore, it is important to build the capacity of road safety researchers, especially transportation engineers, in LMICs in the use of high‐quality epidemiological study designs.

There has been a strong tendency in global advocacy efforts to claim that we already know what works in road safety but that LMICs are failing to implement proven interventions. This EGM highlights that this is largely not true. In fact, as we highlight above, the main issues in road safety that are specific to LMICs have received little systematic attention. While there is strong evidence that protective equipment (e.g., helmets and seatbelts) reduce injuries, even for these there is little evidence on what interventions result in large‐scale increases in their use in LMICs. Therefore, it is important that international development agencies and philanthropic organizations that are engaged in global road safety advocacy efforts emphasize the need for new knowledge on road safety in LMICs. Likewise, it is critical for national and international agencies that fund research and development activities to prioritize road safety research, and use this EGM in setting their research agendas.

### 8.2 Recommendations

The main recommendations from the findings of this EGM are:

- International actors engaged in global road safety advocacy should emphasize the need for new research on road safety interventions especially those that target issues specific to LMICs.
- National and international agencies that fund research and development in LMICs should prioritize road safety and use this EGM to guide their research agendas.
- This EGM finds that investments need to be made in developing the capacity of LMIC engineers and researchers to use epidemiological study designs for evaluating road safety interventions. Relatedly, there is a need to develop capacity to conduct high quality systematic reviews, which includes enabling access of LMIC researchers to online abstract and citation databases.
- Researchers should use this EGM for conducting systematic reviews of interventions for which there are many impact evaluations. Using the EGM database as a starting point can substantially reduce the effort required in searching and screening the literature.
- This EGM should be made a living review so that it functions as a perpetual resource for researchers and policy makers for finding accessible summaries of the road safety literature.

## CONTRIBUTIONS OF AUTHORS

### EGM authors

Dr Rahul Goel – Primary author; subject matter expert human factors and legal and institutional framework; Study screening; Study inclusion criterion.

Prof Geetam Tiwari – Primary author; subject matter expert Road design, infrastructure and traffic control; Study screening.

Dr. Mathew Varghese – Primary author; subject matter expert Pre‐crash and pre‐hospital care.

Prof Kavi Bhalla – Primary author; subject matter expert Vehicle factors and protective devices; Study screening; Study inclusion criterion.

Prof Girish Agrawal – Tertiary author; subject matter expert human factors and legal and institutional framework.

Ms. Guneet Saini – Secondary author; Study data collection; Study screening; Study coding.

Mr. Abhaya Jha – Secondary author; Study data collection; Study screening; Study coding.

Mr. Denny John – Tertiary author; Study inclusion criterion.

Dr. Ashrita Saran – Tertiary author; Quality appraisal of studies.

Dr. Howard White – Tertiary author; Study screening; Study inclusion criterion.

Prof Dinesh Mohan – Primary author; subject matter expert human factors and Legal and institutional framework; Study screening.

### ROLES AND RESPONSIBILITIES

**Content:** DM, GT, KB, MV, GA and RG are members of Independent Council for Road Safety International (ICoRSI) and have been working on transport research and road safety interventions for many years.

**EGM methods:** HW as CEO provides technical and strategic support for the development of EGM in Campbell library. Previously, he has initiated and led the development of EGM during his association with 3ie. DJ is currently co‐author of 3 ongoing EGM registered with Campbell library. AS was lead author of a review of evidence maps, and is leading several on‐going maps, as well as conducting many training workshops on constructing evidence maps.

**Information retrieval:** DJ and AS, in consultation with HW, and other authors will provide information retrieval expertise for the EGM. AJ and GS conducted information retrieval with support from DM, GT, KB, RG and MV.

**Report:** DM, RG, KB, GT, MV, AJ, GA and AS wrote the report.

## DECLARATIONS OF INTEREST

RG, GT, MV, KB, and DM have conducted primary studies on the topic of road safety.

### PLANS FOR UPDATING THE EGM

RG, GT, MV and KB will be responsible for updating EGM every 2 years.

## DIFFERENCES BETWEEN PROTOCOL AND REVIEW

### Intervention criterion

In the intervention category of human factors, we added an additional sub‐category called pedestrian, which includes pedestrian‐related interventions not included in any other intervention categories (e.g., protective clothing).

### Study design

In the protocol, study design categories were based on the commonly used methods in public health literature. In the EGM, we used a different set of categories based on the methods that are prevalent in traffic injury literature. Coding tool also was updated this is reflected in Supporting Information: Appendix B

### Search strategy

In addition to Safety Cube and Road safety handbook, we added Cochrane Injury reviews. Grey literature studies were not searched through string but through a google search.

## SOURCES OF SUPPORT

**Internal sources**

- Independent Council for Road Safety International (ICORSI), India
**External sources**
- National Institutes of Health, USA grant R21 TW010823‐01A1

## Supporting information

Supporting information.
