# Supplementary material for: Effectiveness of road safety interventions: An evidence and gap map
Source: Campbell Syst Rev. 2024 Jan 3;20(1):e1367. doi: 10.1002/cl2.1367 (PMC10765170; doi:10.1002/cl2.1367)
Supplement: Supplementary file 1 — Supporting information. [file CL2-20-e1367-s001.docx]

# Appendices

## 1 Appendix A

## APPENDIX A

Search strategy and databases

**A.1 List of databases**

Academic Databases

| Name of Database | URL |
| --- | --- |
| SafetyLit | <https://www.safetylit.org/> |
| PubMed | <https://pubmed.ncbi.nlm.nih.gov/> |
| Web of Science | <https://clarivate.com/webofsciencegroup/solutions/web-of-science/> |
| EASTS | <http://easts.info/search-easts/> |
| TRID | <https://trid.trb.org/> |
| EMBASE | <https://www.embase.com/login> |
| TRANSPORT | Not found |

All the other academic databases mentioned in the protocol were covered by the above listed databases except the TRANSPORT database which could not be found online.

Gray Literature Websites

We accessed the following transport research-related websites in an effort to find studies relevant to the subject area of the EGM. We also back referenced the relevant systematic reviews and impact studies to identify ongoing, published and unpublished studies.

| Organisation | website |
| --- | --- |
| SafetyCube DSS | <https://www.roadsafety-dss.eu/#/>  Filters- Measures |
| SWOV Institute For Road Safety Research | <https://www.swov.nl/en> |
| Australian Road Research Board (ARRB) | [www.arrb.org.au](http://www.arrb.org.au/) |
| National Highway Traffic Safety Administration(NHTSA), USA | <https://www.nhtsa.gov/> |
| Institute of Transport Economics (TOI), Norway | <http://www.toi.no/> |
| Transport Research Laboratory (TRL) ,UK | [http://www.trl.co.uk](http://www.trl.co.uk/) |
| VTI Swedish National Road and Transport Research Institute | [http://www.vti.se](http://www.vti.se/) |
| VTT Finland | <http://www.vtt.fi/indexe.htm> |
| Joint Transport Research Centre’s International Transport Research Documentation (ITRD) database) |  |
| European Transport Safety Council (ETSC) | <https://etsc.eu/> |
| International Research Council On Biomechanics Of Injury (IRCOBI) | <http://ircobi.org/wordpress/proceedings/> |
| Transportation Research Board (TRB) | <http://www.nas.edu/trb/> |
| World Conference on Transport Research Society (WCTR) | <https://www.wctrs-society.com/> |
| Department for International Development (DFID) | [https://www.gov.uk/government/organisations/department-for-international-development](https://www.gov.uk/government/organisations/department-for-international-developmentResearch%3e%3eTopic:%20Transport%3e%3eSub-topic:%20Driving%20and%20road%20transport;%20road%20infrastructure;%20transport%20accessibility%20and%20mobility;%20transport%20planning;%20local%20transport)  [Research>>Topic: Transport>>Sub-topic: Driving and road transport; road infrastructure; transport accessibility and mobility; transport planning; local transport](https://www.gov.uk/government/organisations/department-for-international-developmentResearch%3e%3eTopic:%20Transport%3e%3eSub-topic:%20Driving%20and%20road%20transport;%20road%20infrastructure;%20transport%20accessibility%20and%20mobility;%20transport%20planning;%20local%20transport) |
| Danish Council for Road Safety Research | <https://www.trm.dk/en/publications/> |
| the Organisation for Economic Co-operation and Development’s (OECD) | [https://www.itf-oecd.org/documents/Road/11?f%5B0%5D=field_theme_tax%3A1&f%5B1%5D=field_category_tax%3A11](https://www.itf-oecd.org/documents/Road/11?f%5B0%5D=field_theme_tax%3A1&f%5B1%5D=field_category_tax%3A11Filters-%20Topic:%20Road%3e%3e%20Subject:%20Safety,%20Security%20and%20Health%3e%3e%20Content%20type:%20Reports) [Filters- Topic: Road>> Subject: Safety, Security and Health>> Content type: Reports](https://www.itf-oecd.org/documents/Road/11?f%5B0%5D=field_theme_tax%3A1&f%5B1%5D=field_category_tax%3A11Filters-%20Topic:%20Road%3e%3e%20Subject:%20Safety,%20Security%20and%20Health%3e%3e%20Content%20type:%20Reports) |
| The Insurance Institute for Highway Safety (IIHS) | <http://iihs.co.in/> |

**A.2 Search hits and Date of Search**

Academic Databases

| Name of Databases | Search Date | Hits |
| --- | --- | --- |
| SafetyLit | August 19 ,2019 | 70237 |
| Pubmed | November 28, 2019 | 15051 |
| Web of Science | November 28, 2019 | 30469 |
| EASTS | December 2, 2019 | 2631 |
| TRID | December 10, 2019 | 25617 |
| EMBASE | December 17, 2019 | 20095 |

Gray Literature Search

| Organisation | Total Studies | Searched studies | Included studies | Search Date | Remarks |
| --- | --- | --- | --- | --- | --- |
| SafetyCube DSS | References of 126 systematic reviews | 613 | 150 | March 19, 2020 | The database was back-referenced in search of relevant studies. Besides the 150 studies included from the references, 87 of 126 systematic reviews fulfilled the eligibility criteria for inclusion and are included in the map manually. |
| SWOV Institute For Road Safety Research | - | - | - | June 3, 2020 | Covered in academic databases, SafetyCube project database and systematic reviews back-referencing |
| Australian Road Research Board (ARRB) | - | - | - | June 3, 2020 | Covered in academic databases, SafetyCube project database and systematic reviews back-referencing |
| National Highway Traffic Safety Administration(NHTSA), USA | - | - | - | June 3, 2020 | Covered in academic databases, SafetyCube project database and systematic reviews back-referencing |
| Institute of Transport Economics (TOI), Norway | - | - | - | June 3, 2020 | Covered in academic databases, SafetyCube project database and systematic reviews back-referencing |
| Transport Research Laboratory (TRL) ,UK | - | - | - | June 3, 2020 | Covered in academic databases, SafetyCube project database and systematic reviews back-referencing |
| VTI Swedish National Road and Transport Research Institute | - | - | - | June 3, 2020 | Covered in academic databases, SafetyCube project database and systematic reviews back-referencing |
| VTT Finland | - | - | - | June 3, 2020 | Covered in academic databases, SafetyCube project database and systematic reviews back-referencing |
| Joint Transport Research Centre’s International Transport Research Documentation (ITRD) | - | - | - | June 3, 2020 | Covered in academic databases, SafetyCube project database and systematic reviews back-referencing |
| European Transport Safety Council (ETSC) | 182 | 182 | 0 | June 3, 2020 | Covered in academic databases, SafetyCube project database and systematic reviews back-referencing |
| International Research Council On Biomechanics Of Injury (IRCOBI) | - | - | - | June 3, 2020 | Covered in academic databases, SafetyCube project database and systematic reviews back-referencing |
| Transportation Research Board (TRB) | - | - | - | June 3, 2020 | Covered in academic databases, SafetyCube project database and systematic reviews back-referencing |
| World Conference on Transport Research Society (WCTR) | 4901 | 4901 | 2 | June 3, 2020 | Covered in academic databases, SafetyCube project database and systematic reviews back-referencing |
| Department for International Development (DFID) | 1047 | 1047 | 2 | June 3, 2020 | Covered in academic databases, SafetyCube project database and systematic reviews back-referencing |
| Danish Council for Road Safety Research | 74 | 74 | 0 | June 3, 2020 | Covered in academic databases, SafetyCube project database and systematic reviews back-referencing |
| the Organisation for Economic Co-operation and Development’s (OECD) | 56 | 56 | 0 | June 3, 2020 | Covered in academic databases, SafetyCube project database and systematic reviews back-referencing |
| The Insurance Institute for Highway Safety (IIHS) | - | - | - | June 3, 2020 | Covered in academic databases, SafetyCube project database and systematic reviews back-referencing |

**A.3 Search Strings**

All the databases were searched using the following keyword search strategy:

| #1 | (crash* OR collision* OR accident* OR traffic OR road* OR street* OR highway* OR freeway* OR walkway* OR expressway* OR crosswalk* OR vehicle*) OR (accident, traffic[MeSH Terms]) |
| --- | --- |
| #2 | (Helmet* OR "protect* device*" OR cloth* OR conspicu* OR visib* OR fluorescen* OR 3M OR scotchlite OR Vest OR equipment OR leather OR glove* OR boot* OR jacket* OR trouser* OR “one piece suit” OR armor OR airbag*OR ABS OR AEB OR brak* OR TCS OR light* OR belt* OR underride OR underrun OR guard* OR seat OR submarining OR warning* OR speed adapt* OR cruise control OR assist* OR alcolock* OR monitor* OR regulat* OR bumper OR bonnet OR airbag*) AND (vehicle* OR automobile* OR taxi* OR car* OR motor* OR “power* two wheeler*” OR bicycl* OR cycl* OR bike* OR rider* OR moped* OR scooter* OR bus* OR truck* OR HGV OR tractor* OR trailer* OR “single unit” OR (motor vehicles[MeSH Terms])) |
| #3 | (extricat* OR extract* OR remov* OR rescue OR entrap* OR evacuat* OR release OR “platinum ten” OR “golden hour”) AND (occupant* or driv* or vehicle* OR automobile* OR taxi* OR car* OR motor* OR “power* two wheeler*” OR bicycl* OR cycle* OR cyclist* OR bike* OR rider* OR moped* OR scooter* OR bus* OR truck* OR HGV OR tractor* OR trailer* OR “single unit” OR (motor vehicles[MeSH Terms])) |
| #4 | (((recidivis* OR Screen* OR renew* OR assess* OR evaluat*) AND (driv* OR vehicle* OR automobile* OR taxi* OR car OR motor* OR “power* two wheeler*” OR cycle* OR cyclist* OR bike* OR rider* OR moped* OR scooter* OR bus* OR truck* OR HGV OR tractor* OR trailer* OR “single unit” OR (motor vehicles[MeSH Terms]))) |
| #5 | ((belt* OR buckle* OR helmet* OR protect* OR phone* OR mobile* OR DUI OR DUID OR “driv* under influence” OR alcohol OR “drink driv*” OR “drunk driv*” OR “drug impair*” OR “drugged driv*”) AND (campaign* OR awareness OR information OR advertis* OR program* OR educat*) |
| #6 | (((speed* OR “red light*” OR camera* OR “drink driving” OR “drunk driving” OR drug* OR “seat belt*” OR DUI OR DUID OR helmet* OR phone* OR "vehicle inspection")) AND (Enforc* OR law* OR rule* OR rehabilitation OR program* OR (enforcement, law[MeSH Terms])))) |
| #7 | ((Pric* OR tax OR insurance OR law) AND (vehicle* OR automobile* OR motor* OR road* OR street* OR congestion* OR fuel OR parking)) |
| #8 | Sanction* OR penalt* OR merit point* OR demerit point* OR prison* OR educat* OR ((licens* OR licenc* OR (license[MeSH Terms])) AND (suspen* OR revocation OR cancel* OR disqualif* OR graduate driv* OR automobile driver examination[MeSH Terms])) |
| #9 | (lane* OR centerline OR centreline OR rumble OR bump* OR ramp* OR shoulder* OR guardrail* OR barrier* OR clearance OR illumina* OR lumin* OR blackspot* OR cushion* OR calming OR footpath* OR footbridge* OR tunnel* OR workzone* OR “work zone*” OR toll* OR sidewalk* OR hotspot* OR motorcycl* OR cyclist* OR pedestrian* OR narrow* OR curb* OR kerb* OR hump* OR island* OR signage* OR crosswalk* OR marking* OR delineat* OR chevron* OR camber OR junction* OR intersection* OR roundabout* OR grade* OR interchange* OR woonerf*) |
| #10 | (#2 OR #3 OR #4 OR #5 OR #6 OR #7 OR #8 OR #9) AND #1 |

## 2 Appendix B

## APPENDIX B

Coding Tool

| Category |  | Answer |
| --- | --- | --- |
| Descriptive information | Title | Open answer |
|  | Author citation | Open answer |
|  | Publication Date | Open answer |
|  | URL | Open answer |
|  | Volume no | Open answer |
|  | Issue no | Open answer |
| Geographical information | World Bank region | - South Asia - Sub-Saharan Africa - East Asia and Pacific - Europe and Central Asia - Latin America and Caribbean - Middle East and North Africa - North America |
|  | Country | - Low income - Lower Middle income - Upper Middle income - High Income Countries |
| Study design |  | - Before-After with control - Case-Control - Cross-Section analysis - Experiments - Time Series with control - Systematic Review |
| Population |  | ROAD USER   - Pedestrian - Driver - Passenger   VEHICLE   - Powered two-wheeler (PTW) - Car - Bus - Truck - Other Motorized vehicles - All Motorized vehicles - Bicycle - Other Non-Motorized vehicles |
| Filters |  | AGE   - All age - 0-5 years - 6 years -Licensing Age - Licensing Age – 65 years - 65 years   ROAD TYPE   - All Road Types - Urban open access roads - Urban restricted access roads - Rural open access roads - Rural restricted access roads |
| Intervention |  | HUMAN FACTORS   - Enforcement - Sanctions and Penalties - Driver Training and Licensing - Road user education, awareness building and public campaigns - Pedestrian   VEHICLE FACTORS AND PROTECTIVE DEVICES   - Bicycle - Powered Two-wheeler - Bus - Truck - Other Vehicles (excluding Car)   ROAD DESIGN, INFRASTRUCTURE AND TRAFFIC CONTROL   - Road design and urban form - Protective Infrastructure - Speed Control Infrastructure - Bicycle, PTW and Pedestrian Paths - Traffic Control and Junctions - Work zones and tollbooths   POST-CRASH PRE-HOSPITAL CARE   - Extrication - Ambulances (Road and helicopter) (including equipment) - Level of medical personnel - First aid training of bystanders - Stay and play vs Scoop and run - Time to hospital - Drugs and medications   LEGAL AND INSTITUTIONAL FRAMEWORK   - Pricing - Safety Institutions - Motor Vehicle Insurance - Laws and policy |
| Outcome |  | PRIMARY OUTCOME   - Fatal crashes - Non-fatal injury crashes   INTERMEDIATE OUTCOME   - Change in use of seat belts - Change in use of helmets - Change in Speed - Change in alcohol/drug use |
